# Epidemiology of herpes simplex virus type 1 in Canada: systematic review, meta-analyses, and meta-regressions

**DOI:** 10.3389/fpubh.2023.1118249

**Published:** 2023-07-14

**Authors:** Sawsan AlMukdad, Manale Harfouche, Uzma S. Farooqui, Lana Aldos, Laith J. Abu-Raddad

**Affiliations:** ^1^Infectious Disease Epidemiology Group, Weill Cornell Medicine-Qatar, Cornell University, Qatar Foundation – Education City, Doha, Qatar; ^2^World Health Organization Collaborating Centre for Disease Epidemiology Analytics on HIV/AIDS, Sexually Transmitted Infections, and Viral Hepatitis, Weill Cornell Medicine-Qatar, Cornell University, Qatar Foundation – Education City, Doha, Qatar; ^3^Department of Population Health Sciences, Weill Cornell Medicine, Cornell University, New York, NY, United States; ^4^Department of Public Health, College of Health Sciences, Member of QU Health, Qatar University, Doha, Qatar

**Keywords:** herpes, genital ulcer disease, seroprevalence, prevalence, meta-analysis, meta-regression, Canada

## Abstract

**Background:**

The objective of this study was to characterize herpes simplex virus type 1 (HSV-1) epidemiology in Canada.

**Methods:**

HSV-1 publications as recent as December 6, 2021 were systematically reviewed, synthesized, and reported following PRISMA guidelines. Meta-analyses and meta-regressions were conducted.

**Results:**

HSV-1 measures were extracted from 22 studies and included 32 overall seroprevalence measures (79 stratified), 2 overall proportions of HSV-1 detection in clinically diagnosed genital ulcer disease (2 stratified), and 8 overall proportions of HSV-1 detection in laboratory-confirmed genital herpes (27 stratified). Pooled mean seroprevalence was 19.1% [95% confidence interval (CI): 12.6–26.4%] among healthy children and 51.4% (95% CI: 47.3–55.5%) among healthy adults. Pooled mean seroprevalence among healthy general populations increased with age, with the lowest being 35.7% (95% CI: 29.1–42.6%) among individuals <20 years of age, and the highest being 70.0% (95% CI: 54.8–83.2) among individuals ≥40 years. Seroprevalence increased by 1.02-fold (95% CI: 1.01–1.04) per year. Pooled mean proportion of HSV-1 detection in genital ulcer disease was 30.8% (95% CI: 12.6–52.8%). Pooled mean proportion of HSV-1 detection in genital herpes was 37.4% (95% CI: 29.5–45.6%) and was highest in women and in young persons. Proportion of HSV-1 detection in genital herpes increased by 1.04-fold (95% CI: 1.00–1.08) per year.

**Conclusions:**

HSV-1 epidemiology in Canada appears to be shifting toward less oral acquisition in childhood and more genital acquisition in adulthood, particularly among youth. Both HSV-1 seroprevalence and proportion of HSV-1 detection in genital herpes are increasing with time.

## Introduction

Herpes simplex virus type 1 (HSV-1) infection is typically acquired orally during childhood (1). HSV-1 infection is lifelong and predominantly asymptomatic (2, 3). Yet, the infection can lead to severe neurological, corneal, or mucocutaneous complications (1, 4). Evidence suggests a shift in the historical pattern of HSV-1 epidemiology in Western countries, with declining oral HSV-1 acquisition in childhood, but increasing genital acquisition among young persons, mostly through oral sex (5–10). Considering the disease burden and changing epidemiology of this infection, the World Health Organization (WHO) and global partners are leading initiatives to enhance our understanding of the epidemiology of this virus and to develop a vaccine that protects against its acquisition (9, 11, 12).

Despite HSV-1 epidemiology being well characterized in the United States (5, 7, 13) and Western Europe (14), the epidemiology of this infection remains inadequately understood in Canada. Accordingly, we conducted a comprehensive systematic review to characterize HSV-1 epidemiology in this country. The study aimed to characterize HSV-1 trends and patterns for the purpose of informing policy, programming, and resource allocation, as well as to address the disease burden of this infection, an infection for which there are at present no specific prevention and control strategies in place in Canada.

The study implemented an established analytical approach that has been developed, tested, and refined over years of investigation and applications for a range of infections (15–20). Meta-analytical methods were employed to estimate HSV-1 antibody prevalence (seroprevalence), and proportions of HSV-1 detection in clinically diagnosed genital ulcer disease (GUD) and in laboratory-confirmed genital herpes. Meta-regressions were conducted to investigate associations and overall temporal trends over the study timeframe for each of HSV-1 seroprevalence and proportion of HSV-1 detection in genital herpes. While ideally trends in seroprevalence are best established through repeated cross-sectional surveys on the same population over a long time horizon, such data do not exist for HSV-1 infection except in one country, the United States, through the NHANES surveys done for over four decades (7, 13). It is challenging to justify such costly surveys for HSV-1 infection worldwide. Therefore, our study addresses a gap in evidence for Canada that otherwise could not have been filled.

## Materials and methods

The methodology used in this study was based on that developed in a series of published systematic reviews investigating HSV-1 and HSV-2 epidemiology in other regions and countries (14–17, 21–27). Therefore, no study protocol was registered in PROSPERO for this specific study. The methodology is described in Box 1 and is summarized below.

BOX 1 **Description of the methodology for this study.**

**Table:** 

Methodology	Description
Data source and search strategy	Search conducted on December 6, 2021 in PubMed and Embase. Search strategies included exploded MeSH/Emtree terms and broad terms with no language or time restrictions.
Study selection and inclusion and exclusion criteria	Search results were imported into the reference manager Endnote (Thomson Reuters, United States). Screening was performed in four stages: Duplicate publications were identified and excluded. Titles and abstracts were screened for relevant and potentially relevant publications. Full texts of relevant and potentially relevant publications were retrieved and screened for relevance. Bibliographies of relevant publications and reviews were checked for additional potentially relevant publications. Inclusion criteria were any publication, with a minimum sample size of 10, reporting primary data on any of the following outcome measures: HSV-1 antibody seroincidence as detected by a type-specific diagnostic assay. HSV-1 seroprevalence as detected by a type-specific diagnostic assay. Proportion of HSV-1 in clinically diagnosed GUD as detection by standard viral detection and subtyping methods. Proportion of HSV-1 in laboratory-confirmed genital herpes (as opposed to HSV-2), as detection by standard viral detection and subtyping methods. Exclusion criteria were: Case reports, case series, reviews, editorials, commentaries, and qualitative studies. Measures reporting seroprevalence in infants <6 months old as their antibodies can be maternal in origin. In this study, the term “publication” refers to a document reporting one or several outcome measures. “Study” or “measure” refers to a specific outcome measure and its details.
Data extraction and data synthesis	Extracted variables included: author(s), publication title, year(s) of data collection, year of publication, country of origin, country of survey, city, study site, study design, study sampling procedure, study population and its characteristics (e.g., sex and age), sample size, HSV-1 outcome measures, and diagnostic assay. Stratification hierarchy for seroprevalence in descending order of preference was population type, age bracket (children versus adults), and age group: Population type classified as: Healthy general populations: healthy populations such as blood donors, pregnant women, and outpatients with minor health conditions. Clinical populations: any population with a major clinical condition, or a condition related (potentially) to HSV-1 infection. Other populations: other populations not satisfying above definitions, or populations with an undetermined risk of acquiring HSV-1, such as HIV-positive patients, sex workers, men who have sex with men, and prisoners. Age bracket classified as: Children: ≤15 years old individuals. Adults: >15 years old individuals. Age group classified as (groups optimized to best fit reported data): <20 years old. 20–29 years old. 30–39 years old. ≥40 years old. Mixed age bands. Stratification hierarchy for GUD and genital herpes included genital herpes episode status and study site: Genital herpes episode status classified as: Primary genital herpes. Recurrent genital herpes. Study site stratification classified as: Hospital. Sexually transmitted disease clinic.
Quality assessment	The Cochrane-informed approach for risk of bias assessment included: Study’s precision classification into low versus high based on the sample size (<100 versus ≥ 100). Study’s appraisal into low versus high risk of bias was determined using two quality domains: Sampling method: probability-based versus non-probability based. Response rate: ≥80% versus < 80% or unclear.
Meta-analyses	Meta-analyses were conducted using DerSimonian-Laird random-effects models with inverse variance weighting. The variance of each outcome measure was stabilized using the Freeman-Tukey arcsine square-root transformation. Pooled means of HSV-1 seroprevalence were estimated by population type, age bracket, age group, sex, year of publication category, and year of data collection category. Overall pooled proportion of HSV-1 detection in clinically diagnosed GUD cases was estimated. Pooled proportion of HSV-1 detection in laboratory-confirmed genital herpes cases was estimated by age group, sex, year of publication category, and year of data collection category. Heterogeneity assessment was based on three complementary metrics: Cochran’s Q statistic to assess existence of heterogeneity in effect size (*p-*value<0.1 indicated heterogeneity). I^2^ heterogeneity measure to assess the percentage of between-study variation in effect size that is due to actual differences in effect size rather than chance. Prediction interval to describe the distribution of true outcome measures around the pooled mean.
Meta-regressions	Univariable and multivariable random-effects meta-regression analyses using log-transformed proportions were carried out to identify predictors of HSV-1 seroprevalence and proportion of HSV-1 detection in laboratory-confirmed genital herpes. Factors in the univariable model with a *p-*value<0.1 were included in the multivariable analysis. Factors in the multivariable model with a *p-*value≤0.05 were deemed to be significant predictors. Variables included in the univariable meta-regression model for HSV-1 seroprevalence were: Age bracket. Age group. Sex. Population type. Assay type (western blot, ELISA, and neutralization). Sample size. Sampling method. Response rate. Year of data collection. Year of data collection category (≤2000; >2000). Variables included in the univariable meta-regression model for proportion of HSV-1 detection in laboratory-confirmed genital herpes were: Age group. Sex. Year of data collection. Year of data collection category (≤2000; >2000).

ELISA, enzyme-linked immunosorbent type-specific assay; GUD, genital ulcer disease; HSV-1, herpes simplex virus type 1; HSV-2, herpes simplex virus type 2.

### Data sources, search strategy, study selection, and eligibility criteria

HSV-1 publications were systematically reviewed as informed by the Cochrane Collaboration Handbook (28), and the results were reported following the Preferred Reporting Items for Systematic Reviews and Meta-analyses (PRISMA) guidelines (29, 30) (Supplementary Table S1). Search strategies are detailed in Supplementary Table S2 and were based on those developed in a series of published HSV-1 and HSV-2 systematic reviews (14–17, 21–27). The systematic literature search was conducted using PubMed and Embase databases, up until December 6, 2021. MeSH/Emtree terms, keywords, and broad search criteria were applied with no year or language restrictions to broaden the search scope and to ensure inclusivity (Supplementary Table S2). In addition, we searched institutional websites of Canadian public health authorities to identify potentially relevant reports including data not published in the scientific literature (Supplementary Table S2). Screening processes, inclusion criteria, and exclusion criteria are described in Box 1. Titles and abstracts of all citations were screened independently twice for relevant and potentially relevant publications, with the screening split among three reviewers (SM, UF, and LA).

### Data extraction, synthesis, and quality assessment

Each of data extraction and double extraction from eligible studies were performed independently twice, with the extraction split among four reviewers (SM, MH, UF, and LA). Discrepancies in data extraction were settled by consensus, including also LJA, and, if needed, by contacting authors. *A priori* determined list of variables was used to extract data (Box 1). A quality assessment of the sensitivity and specificity of HSV-1 diagnostic assays was performed, given their known limitations, including the potential cross-reactivity with HSV-2 antibodies (31–35). This was done with the support of Professor Rhoda Ashley-Morrow of the University of Washington, an expert advisor in HSV-1 diagnostic methods. Only studies that utilized valid and reliable type-specific assays, with no potential for cross-reactivity with HSV-2, were included.

Included studies were evaluated for precision and risk of bias (ROB) as informed by the Cochrane approach (Box 1). Study precision was classified as either low or high, depending on whether the overall sample size was <100 or ≥ 100. Two quality domains were used to distinguish low versus high ROB: sampling method (probability-based versus non-probability-based) and response rate (≥80% versus < 80% or unclear) (Box 1).

Both overall measures and stratified measures were extracted from relevant studies (Box 1). Since our aim was to characterize the natural heterogeneity that exists in HSV-1 epidemiology, such as the variation in HSV-1 seroprevalence between children and adults, measures were extracted and stratified by key epidemiological factors known to affect the natural epidemiology of this infection (14, 17, 23, 24, 26, 27). Meta-regression analyses were further conducted on these stratified measures to estimate effects of these epidemiological factors on both HSV-1 seroprevalence and proportion of HSV-1 detection in genital herpes.

### Meta-analyses

Meta-analyses were conducted using the DerSimonian-Laird random-effects model (36) with the Freeman-Tukey double arcsine transformation (37), after ensuring the transformation’s applicability given available data in this systematic review (38). The meta-analyses were used to obtain pooled mean estimates for HSV-1 seroprevalence and proportions of HSV-1 detection in GUD and in genital herpes (Box 1). These pooled estimates are meant to provide an average summary measure of the actual measures that exist in the population, as an overall measure and by specific factors or timeframes. The meta package (39) was used to perform these analyses in R version 4.0.4 (40).

### Meta-regressions

Meta-regression analyses on log transformed outcome measures (seroprevalence and proportion of HSV-1 detection in genital herpes) were conducted in Stata/SE version 16 using the metareg package (41) to investigate between-study heterogeneity, potential associations, and overall temporal trends for HSV-1 seroprevalence and proportion of HSV-1 detection in genital herpes (Box 1). A linear relationship was assumed between the log transformed outcome measures and each of the independent variables $\left(y=\beta_{0}+\underset{i=1}{\sum }\beta_{i}x_{i}\right)$ . Back transformation was used to estimate the adjusted relative risks (aRR).

## Results

### Search results and scope of evidence

**Figure 1:**
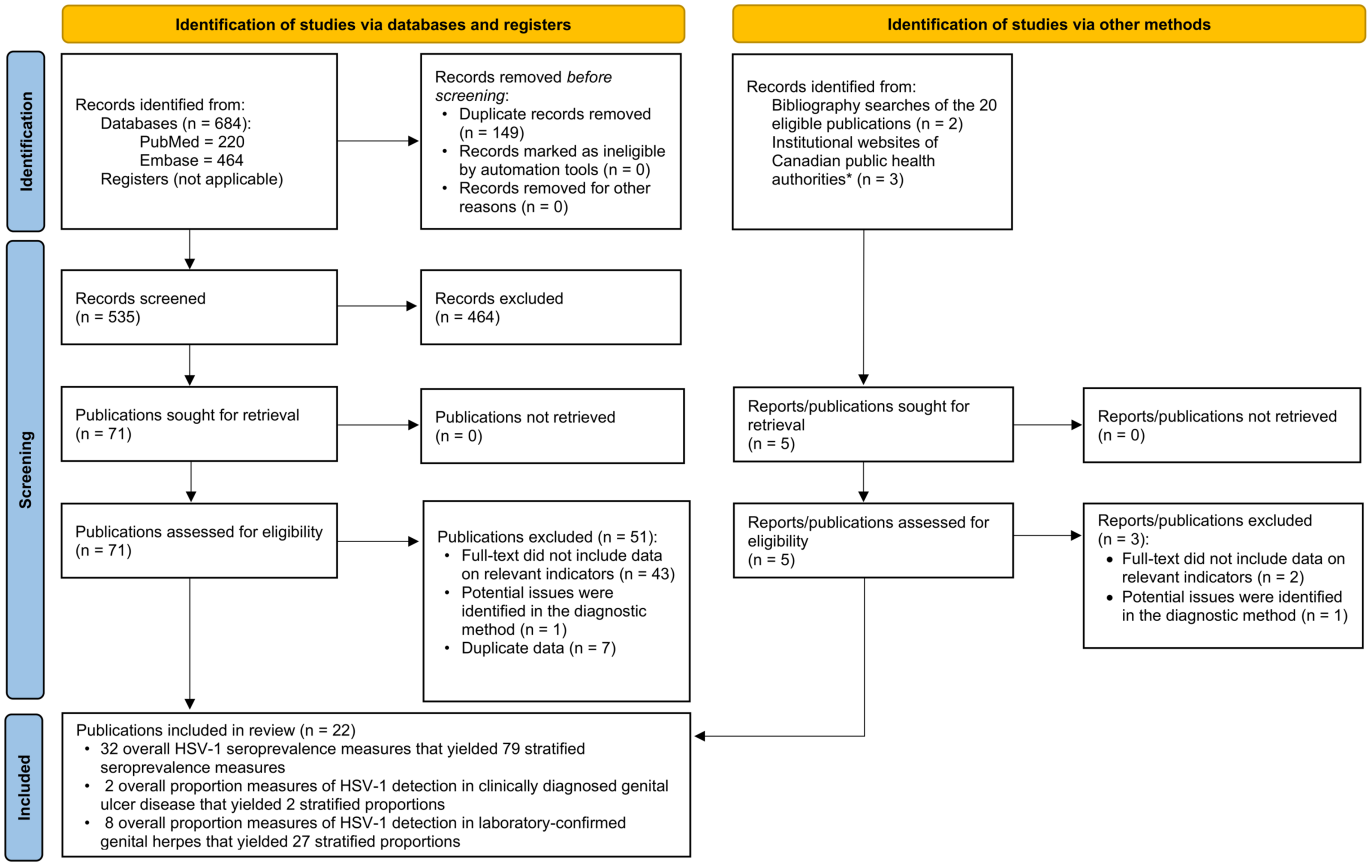
Flow diagram of article selection for the systematic review of HSV-1 infection in Canada, per PRISMA guidelines (30). *List of institutional websites of Canadian public health authorities are in Supplementary Table S2. HSV-1, Herpes simplex virus type 1.

The study selection process per PRISMA guidelines is summarized in Figure 1. The search identified 684 publications (220 in PubMed and 464 in Embase), of which 20 proved relevant. Screening of bibliographies of relevant publications identified two additional relevant articles (42, 43). In total, 22 publications met the inclusion criteria. Extracted HSV-1 measures included 32 overall seroprevalence measures (79 stratified), 2 overall proportions of HSV-1 detection in clinically diagnosed GUD (2 stratified), and 8 overall proportions of HSV-1 detection in laboratory-confirmed genital herpes (27 stratified). No studies on HSV-1 seroincidence were identified. Publications and reports that were excluded after full-text screening from both databases and institutional websites of public health authorities in Canada are shown in Supplementary Table S7.

### Seroprevalence overview

Overall HSV-1 seroprevalence measures are listed in Supplementary Table S3. Most studies were published in 2005 or before (*n* = 19; 59.4%) and were conducted using convenience sampling (*n* = 23; 71.9%). Stratified HSV-1 seroprevalence measures for different populations and subpopulations are summarized in Table 1.

**Table 1:** Pooled mean estimates for HSV-1 seroprevalence in Canada.

Populations	Outcome measures^*^	Samples	HSV-1 seroprevalence		Pooled mean HSV-1 seroprevalence	Heterogeneity measures		
	Total n	Total N	Range	Median	Mean (95% CI)	Q^†^ (*p*-value)	I²^‡^ (%) (95% CI)	Prediction interval^§^ (%)
Healthy general populations								
Age bracket								
Children Adults	4	134	10.7–28.6	19.2	19.1 (12.6–26.4)	2.7 (*p* = 0.438)	0.0 (0.0–84.7)	6.3–36.0
	59	7,769	12.5–89.0	52.1	51.4 (47.3–55.5)	592.1 (*p* < 0.001)	90.2 (88.1–91.9)	23.3–79.1
Sex								
Women Men	54	6,924	10.7–89.0	52.1	50.7 (45.9–55.4)	552.8 (*p* < 0.001)	90.4 (88.3–92.1)	19.4–81.7
	9	979	28.0-63.0	43.0	42.3 (33.7–51.1)	59.2 (*p* < 0.001)	86.5 (76.4–92.3)	14.5–73.0
Age group								
<20 years 20-29 years 30–39 years ≥40 years Mixed	15	2,677	10.7–55.0	32.0	35.7 (29.1–42.6)	83.0 (*p* < 0.001)	83.1 (73.5–89.3)	12.1–63.7
	13	2,519	29.0–68.0	53.0	52.5 (46.5–58.4)	70.9 (*p* < 0.001)	83.1 (72.4–89.6)	30.0–74.4
	8	783	56.0–75.0	67.0	64.4 (59.6–69.0)	11.7 (*p*= 0.112)	40.0 (0.0–73.5)	52.3–75.6
	4	382	55.0–89.0	67.3	70.0 (54.8–83.2)	19.5 (*p* < 0.001)	84.6 (61.5–93.8)	6.6–100.0
	23	1,542	12.5–87.6	46.4	47.1 (39.9–54.4)	228.9 (*p* < 0.001)	90.4 (86.9–92.9)	16.7–78.6
Year of publication category
≤2005 >2005	59	4,919	10.7–89.0	52.0	48.8 (44.4–53.1)	395.1 (*p* < 0.001)	85.3 (81.8–88.2)	19.5–78.5
	4	2,984	41.0–87.6	49.3	57.9 (35.4–78.8)	256.4 (*p* < 0.001)	98.8 (98.2–99.2)	0.0–100.0
Year of data collection category
≤2000 >2000	31	2,572	10.7–65.2	46.4	44.5 (38.8–50.2)	150.5 (*p* < 0.001)	80.1 (72.3–85.6)	17.4–73.4
	32	5,331	28.9–89.0	52.5	53.9 (47.8–60.0)	500.3 (*p* < 0.001)	93.8 (92.2–95.1)	20.9–85.1
All healthy general populations	63	7,903	10.7–89.0	51.0	49.4 (45.1–53.7)	653.7 (*p* < 0.001)	90.5 (88.6–92.1)	19.1–80.0
Clinical populations								
Clinical adults	8	8,796	34.0–75.1	51.5	54.2 (43.3–64.9)	284.1 (*p* < 0.001)	97.5 (96.5–98.3)	17.7–88.2
Other populations								
HIV-positive patients	7	1,317	73.7–90.2	78.1	80.1 (74.5–85.1)	16.3 (*p* = 0.013)	63.1 (16.2–83.7)	62.7–93.2
Men who have sex with men	1^¶^	144	–	–	69.4 (61.2–76.8)	–	–	–

^*^The meta-analyses were based on the 79 stratified HSV-1 seroprevalence measures.

^†^Q: Cochran’s Q statistic is a measure assessing heterogeneity in pooled outcome measures, here HSV-1 seroprevalence.

^‡^I^2^: A measure assessing the magnitude of between-study variation due to true differences in HSV-1 seroprevalence across studies rather than to sampling variation.

^§^Prediction interval: A measure quantifying the 95% interval of the distribution of true HSV-1 seroprevalence around the estimated pooled mean.

^¶^No meta-analysis was done due to the small number of studies (*n* < 3).

CI, confidence interval; HIV, human immunodeficiency virus; HSV-1, herpes simplex virus type 1.

### Pooled mean estimates for HSV-1 seroprevalence

The meta-analyses were based on the 79 stratified HSV-1 seroprevalence measures. Pooled mean seroprevalence for healthy children, with a median age of 9 years, was 19.1% [95% confidence interval (CI): 12.6–26.4%]. In contrast, pooled mean seroprevalence for healthy adults was significantly higher at 51.4% (95% CI: 47.3–55.5%) (Table 1). Pooled mean seroprevalence for clinical adult populations was 54.2% (95% CI: 43.3–64.9%). Pooled mean seroprevalence for HIV-positive patients was 80.1% (95% CI: 74.5–85.1%).

Pooled mean seroprevalence in healthy general populations increased with age from 35.7% (95% CI: 29.1–42.6%) among individuals <20 years of age, followed by 52.5% (95% CI: 46.5–58.4%) in those 20–29 years, 64.4% (95% CI: 59.6–69.0%) in those 30–39 years, and 70.0% (95% CI: 54.8–83.2) in those ≥40 years.

Most meta-analyses showed evidence of heterogeneity (*p*-value<0.001) with wide prediction intervals (Table 1). Most seroprevalence variation was caused by true differences in seroprevalence, as opposed to sampling variation (*I*^2^ > 50%). This affirms the need for meta-regressions to explain this heterogeneity. Forest plots for the meta-analyses by population type classification are shown in Supplementary Figure S1.

### Sources of between-study heterogeneity and predictors of HSV-1 seroprevalence

In the univariable meta-regression analyses for HSV-1 seroprevalence, the following variables were eligible for inclusion in the final multivariable analyses: age bracket, age group, sex, population type, assay type, sample size, sampling method, response rate, and year of data collection (Table 2 and Supplementary Table S4). Because of collinearity between age bracket and age group, and collinearity between year of data collection as a linear term and as a categorical variable, four multivariable models were conducted. The four models analyzed the 79 stratified HSV-1 seroprevalence measures.

**Table 2:** Univariable and multivariable meta-regression analyses for HSV-1 seroprevalence in Canada.

			Outcome measures	Samples	Univariable analysis^*^					Multivariable analyses^*†^			
										Model 1^‡^		Model 2^§^	
			Total n	Total N	*RR* (95% CI)	*p*-value	LR test *p*-value	Adjusted *R*^2^ (%)		*aRR* (95% CI)	*p*-value	*aRR* (95% CI)	*p*-value
Population Characteristics	Age bracket	Children	4	134	1.00	–		<0.001	14.68	1.00	–	–	–
		Adults	75	18,026	2.68 (1.68–4.29)	<0.001				2.15 (1.37–3.38)	0.001	–	–
	Age group	<20	15	2,677	1.00	–		<0.001	36.72	–	–	1.00	–
		20–29	13	2,519	1.40 (1.13–1.73)	0.003				–	–	1.28 (1.11–1.48)	0.001
		30–39	8	783	1.76 (1.38–2.24)	<0.001				–	–	1.57 (1.34–1.84)	<0.001
		≥40	7	441	1.94 (1.50–2.51)	<0.001				–	–	1.65 (1.36–2.00)	<0.001
		Mixed	36	11,740	1.46 (1.21–1.75)	<0.001				–	–	1.79 (1.47–2.18)	<0.001
	Sex	Women	59	7,388	1.00	–		0.006	14.97	1.00	–	1.00	–
		Men	11	1,411	0.92 (0.75–1.13)	0.404				0.74 (0.62–0.88)	0.001	0.76 (0.66–0.86)	<0.001
		Mixed	9	9,361	1.40 (1.12–1.74)	0.003				1.15 (0.85–1.55)	0.355	1.07 (0.78–1.32)	0.498
	Population type	Healthy	63	7,903	1.00	–		<0.001	22.91	1.00	–	1.00	–
		Clinical	8	8,796	1.06 (0.85–1.33)	0.589				0.95 (0.72–1.25)	0.700	0.81 (0.65–1.01)	0.064
		Other	8	1,461	1.56 (1.25–1.95)	<0.001				1.34 (0.98–1.84)	0.063	1.04 (0.82–1.34)	0.728
Study methodology characteristics	Assay type	Western blot	7	3,659	1.00	–		<0.001	16.88	1.00	–	1.00	–
		ELISA	44	13,395	1.16 (0.92-1.46)	0.217				1.22 (0.97–1.54)	0.093	1.23 (1.04–1.44)	0.013
		Neutralization	28	1,106	0.83 (0.64–1.07)	0.146				1.17 (0.81–1.69)	0.407	0.98 (0.75–1.30)	0.909
	Sample size^¶^	<100	3	59	1.00	–		0.039	5.48	1.00	–	1.00	–
		≥100	76	18,101	0.66 (0.45–0.98)	0.039				1.28 (0.86–1.91)	0.220	1.14 (0.77–1.67)	0.513
	Sampling method	Probability based	54	4,523	1.00	–		0.020	5.72	1.00	–	1.00	–
		Non-probability based	25	13,637	1.21 (1.03–1.42)	0.020				1.04 (0.81–1.33)	0.774	0.99 (0.84–1.19)	0.993
	Response rate	<80%	21	7,119	1.00	–		0.001	8.21	1.00	–	1.00	–
		Unclear	58	11,041	1.38 (1.14–1.66)	0.001				1.06 (0.81–1.38)	0.690	1.14 (0.94–1.38)	0.175
Year of data collection			79	18,160	1.01 (1.01–1.02)	<0.001		<0.001	17.91	1.01 (0.99-1.02)	0.136	1.02 (1.01–1.04)	<0.001

^*^The meta-regression analyses were based on the 79 stratified HSV-1 seroprevalence measures.

^†^Two multivariable models were conducted, one including age bracket (children *versus* adults) and one including age group.

^‡^Variance explained by the final multivariable model 1 (adjusted *R^2^*) = 50.27%.

^§^Variance explained by the final multivariable model 2 (adjusted *R^2^*) = 80.12%.

^¶^Sample size denotes the sample size of each study population found in the original publication.

*aRR*, adjusted risk ratio; CI, confidence interval; ELISA, enzyme-linked immunosorbent type-specific assay; HSV-1, herpes simplex virus type 1; *RR*, risk ratio.

The model that included age group, sex, population type, assay type, sample size, sampling method, response rate, and year of data collection as a linear term explained 80.12% of the variation (heterogeneity) in HSV-1 seroprevalence (Table 2). Compared to individuals <20 years of age, seroprevalence was 1.28-fold (95% CI: 1.11–1.48) higher in those 20–29 years, 1.57-fold (95% CI: 1.34–1.84) higher in those 30–39 years, and 1.65-fold (95% CI: 1.36–2.00) higher in those ≥40 years. Men had 0.76-fold (95% CI: 0.66–0.86) lower seroprevalence than women. Seroprevalence increased by 1.02-fold (95% CI: 1.01–1.04) per year.

Compared to studies using western blot as a diagnostic assay, seroprevalence was higher in studies that used enzyme-linked immunosorbent assays (ELISA) (Table 2). There was no evidence for differences in seroprevalence by population type (healthy versus clinical), sample size, sampling method, and response rate. The remaining three multivariable models confirmed similar findings (Table 2 and Supplementary Table S4).

### HSV-1 detection in clinically diagnosed GUD and in laboratory-confirmed genital herpes

Overall proportions of HSV-1 detection in clinically diagnosed GUD and in laboratory-confirmed genital herpes are listed in Supplementary Table S5. Stratified proportions of these measures are summarized in Table 3. In GUD cases (*n* = 2), pooled mean proportion of HSV-1 detection was 30.8% (95% CI: 12.6–52.8%; Table 3).

**Table 3:** Pooled mean proportions of HSV-1 detection in clinically diagnosed genital ulcer disease and in laboratory-confirmed genital herpes in Canada.

Population type	Outcome measures^*^	Samples	Proportion of HSV-1 detection (%)		Pooled proportion of HSV-1 detection (%)	Heterogeneity measures		
	Total n	Total N	Range	Median	Mean (95% CI)	Q^†^ (*p*-value)	I²^‡^ (%) (95% CI)	Prediction interval^§^ (%)
Patients with clinically diagnosed GUD								
All patients with GUD	2^¶^	8,130	20.8–41.8	31.3	30.8 (12.6–52.8)	–	–	–
Patients with laboratory-confirmed genital herpes								
Sex								
Women	10	1,790	4.5–75.8	39.8	41.8 (26.1–58.4)	266.0 (*p* < 0.001)	96.6 (95.2–97.6)	0.1–95.0
Men	9	468	18.4–53.9	32.3	34.0 (25.9–42.6)	26.6 (*p* = 0.001)	70.0 (40.2–84.9)	10.9–61.9
Mixed	8	32,635	1.0–62.6	40.4	36.0 (21.4–52.0)	637.7 (*p* < 0.001)	98.9 (98.6–99.2)	0.0–89.8
Age group								
<30 years	6	1,323	33.7–75.8	60.0	60.0 (46.7–72.6)	67.8 (*p* < 0.001)	92.6 (86.7–95.9)	15.4–96.2
30–39 years	4	524	32.3–49.7	42.7	43.2 (36.7-49.7)	6.0 (*p* = 0.113)	49.7 (0.0–83.4)	20.3–67.7
≥40 years	9	411	4.5–35.5	22.2	21.9 (15.2–29.5)	23.7 (*p* = 0.002)	66.3 (31.6–83.4)	4.4–46.8
Mixed	8	32,635	1.0–62.6	40.4	36.0 (21.4–52.0)	637.7 (*p* < 0.001)	98.9 (98.6–99.2)	0.0–89.8
Year of publication category
≤2005	20	2,458	1.0–75.8	33.0	35.0 (24.9–45.8)	736.1 (*p* < 0.001)	97.4 (96.8–97.9)	0.8–83.9
>2005	7	32,435	33.8–62.6	40.6	43.5 (36.1–51.0)	383.1 (*p* < 0.001)	98.4 (97.8–98.9)	18.9–69.9
Year of data collection category
≤2000	20	2,458	1.0–75.8	33.0	35.0 (24.9–45.8)	736.1 (*p* < 0.001)	97.4 (96.8–97.9)	0.8–83.9
>2000	7	32,435	33.8–62.6	40.6	43.5 (36.1–51.0)	383.1 (*p* < 0.001)	98.4 (97.8–98.9)	18.9–69.9
All patients with genital herpes	27	34,893	1.0–75.8	36.4	37.4 (29.5–45.6)	1,190.5 (*p* < 0.001)	97.8 (97.4–98.2)	4.1–79.9

^*^The meta-analyses were based on the 29 stratified proportions of HSV-1 detection in clinically diagnosed genital ulcer disease and in laboratory-confirmed genital herpes.

^†^Q: Cochran’s Q statistic is a measure assessing heterogeneity in pooled outcome measures, here proportions of HSV-1 detection.

^‡^I^2^: A measure assessing the magnitude of between-study variation due to true differences in proportions of HSV-1 detection across studies rather than to sampling variation.

^§^Prediction interval: A measure quantifying the 95% interval of the distribution of true proportions of HSV-1 detection around the estimated pooled mean.

^¶^No meta-analysis was done due to the small number of studies (*n* < 3). The two study samples were merged to yield one sample size, for which the 95% CI was calculated.

CI, confidence interval; GUD, genital ulcer disease; HSV-1, herpes simplex virus type 1.

The meta-analyses were based on the 27 stratified proportions of HSV-1 detection in genital herpes. The pooled mean proportion of HSV-1 detection in genital herpes was 37.4% (95% CI: 29.5–45.6%; Table 3). Among women (*n* = 10), the pooled mean proportion was 41.8% (95% CI: 26.1–58.4%). Among men (*n* = 9), the pooled mean proportion was 34.0% (95% CI: 25.9–42.6%).

Heterogeneity was evident in most meta-analyses (*p*-value<0.001, *I*^2^ > 50%) and resulted in wide prediction intervals. A forest plot of the meta-analysis for the proportion of HSV-1 detection in laboratory-confirmed genital herpes is shown in Supplementary Figure S2.

### Sources of between-study heterogeneity and predictors of HSV-1 detection in genital herpes

Results of the univariable and multivariable meta-regressions for the proportion of HSV-1 detection in laboratory-confirmed genital herpes are shown in Table 4. The multivariable model explained 84.3% of the variation (heterogeneity) in HSV-1 proportion and included age group, sex, and year of data collection as a linear term (Table 4). The model analyzed the 27 stratified proportions of HSV-1 detection in genital herpes.

**Table 4:** Univariable and multivariable meta-regression analyses for HSV-1 detection in laboratory-confirmed genital herpes in Canada.

		Outcome measures	Samples	Univariable analysis^*^				Multivariable analysis^*†^	
		Total n	Total N	*RR* (95% CI)	*p*-value	LR test *p*-value	Adjusted *R*^2^ (%)	*aRR* (95% CI)	*p*-value
Age group	<30	6	1,323	1.00	–	0.012	68.74	1.00	–
	30–39	4	524	0.72 (0.43–1.19)	0.192			0.71 (0.48–1.04)	0.074
	≥40	9	411	0.42 (0.26–0.68)	0.001			0.42 (0.28–0.62)	<0.001
	Mixed	8	32,635	0.68 (0.45–1.04)	0.072			0.47 (0.32–0.71)	0.001
Sex^‡^	Women	10	1,790	1.00	–	0.780	0.00	1.00	–
	Men	9	468	0.81 (0.43–1.51)	0.488			0.72 (0.51–0.99)	0.046
	Mixed	8	32,635	0.89 (0.48–1.66)	0.700			-^¶^	-^¶^
Year of data collection category^§^	≤2000	20	2,458	1.00	-	0.436	0.00	-	-
	>2000	7	32,435	1.24 (0.71–2.15)	0.436			-	-
Year of data collection		27	34,893	1.05 (0.99–1.10)	0.076	0.076	0.00	1.04 (1.00–1.08)	0.044

^*^The meta-regression analyses were based on the 27 stratified proportions of HSV-1 detection in genital herpes.

^†^Variance explained by the final multivariable model (adjusted *R^2^*) = 84.27%.

^‡^Although the sex variable did not have a statistically significant association with the outcome in the univariable analysis (*p*-value>0.1), it was included in the multivariable analysis because of epidemiological relevance.

^§^Only the linear term of year of data collection was considered in the multivariable analysis since the categorical variable did not have a statistically significant association with the outcome in the univariable analysis (*p*-value>0.1).

^¶^Mixed sex variable was not included in the multivariable model due to collinearity with the mixed age group variable.

*aRR*, adjusted risk ratio; CI, confidence interval; HSV-1, herpes simplex virus type 1; *RR*, risk ratio.

Compared to individuals <30 years of age, the proportion of HSV-1 detection was 0.71-fold (95% CI: 0.48–1.04) lower in those 30–39 years, and 0.42-fold (95% CI: 0.28–0.62) lower in those ≥40 years. Compared to women, the proportion of HSV-1 detection in genital herpes was 0.72-fold (95% CI: 0.51–0.99) lower in men. The proportion of HSV-1 detection in genital herpes increased by 1.04-fold (95% CI: 1.00–1.08) per year.

### Quality assessment

Outcomes of quality assessment are shown in Supplementary Table S6. Twenty-nine studies (90.6%) were of high precision, 9 studies (28.1%) were of low ROB in the sampling method domain, and no studies were of low ROB in the response rate domain. Three (9.4%) studies were of low precision, 23 studies (71.9%) were of high ROB in the sampling method domain, and 5 studies (15.6%) were of high ROB in the response rate domain. No studies were of low ROB in both quality domains, while only one study (3.1%) was of high ROB in both quality domains. For 27 studies (84.4%), the ROB assessment for the response rate domain was “unclear.” Notably, in the meta-regressions for HSV-1 seroprevalence, none of the precision and ROB domains were significantly associated with HSV-1 seroprevalence (Table 2 and Supplementary Table S4).

## Discussion

This study provided a detailed characterization and assessment of HSV-1 epidemiology in Canada. Both HSV-1 seroprevalence and proportion of HSV-1 detection in genital herpes appears to be increasing with time in this country. Two-thirds of youth are approaching sexual debut without being infected orally in childhood; thus, they are at risk of acquiring the infection genitally, through oral-genital sex or genital-genital sex, causing genital herpes (5). As a result, a range of psychosexual adverse outcomes can emerge such as effects on sexual relations and quality of life, depression, anxiety, and low self-esteem (44–47).

The shift in HSV-1 epidemiology from oral to increasingly genital acquisition in Canada resembles that observed in the United States, Western Europe, and Australia and New Zealand (5, 7, 14, 27, 48). This shift particularly affects youth and women, where rates of HSV-1 detection in genital herpes were highest (Table 4). However, unlike the United States and Western Europe (5, 7, 14, 48), HSV-1 seroprevalence is on the rise in Canada, potentially due to an increase in immigration from regions where HSV-1 seroprevalence rates are higher, notably Asian countries. These countries contribute to more than half of the immigrant population arriving in Canada (49). This increase was also observed in Australia, perhaps for a similar reason (27). Nevertheless, the seroprevalence of HSV-1 in Canada is comparable to that in the United States, standing at 58% (5, 7). However, it remains lower than the estimated global HSV-1 seroprevalence, which is estimated using mathematical modeling at 67% (9). In a global context, Canada’s seroprevalence rate is relatively low and significantly below the historical levels of near-universal childhood infection observed in other regions. For instance, Europe reports a seroprevalence of 74% (14), Asia at 77% (17), Australia at 85% (27), Latin America and the Caribbean at 85% (26), the Middle East and North Africa at 89% (23), and Africa at 96% (24).

HSV-1 seroprevalence increases with age, reflecting lifetime cumulative exposure, just as elsewhere (17, 23, 24, 26, 48). Age alone explained one-third of seroprevalence variation (Table 2). Seroprevalence among children was much less than among adults, suggesting, in context of the global epidemiology of this infection and its historical pattern (14, 17, 23, 24, 26, 27), that older cohorts had higher exposure in childhood, compared to the current cohort of children. Seroprevalence among healthy children in Canada, standing at 19%, was found to be comparatively lower than that of Europe (32%) (14), Asia (49%) (17), Latin America and the Caribbean (57%) (26), the Middle East and North Africa (65%) (23), and Africa (69%) (24). Seroprevalence was lower in males than females, a pattern seen elsewhere only in Europe and Australia (14, 27), in contrast to the global pattern (17, 23, 24, 26), in which there are no significant differences in seroprevalence by sex.

HSV-1 (versus HSV-2) detection in genital herpes was high at 37%, a level similar to that observed in the United States (33%) (50), Western Europe (34%) (14), and Australia and New Zealand (31%) (27), but much higher than the level observed in other regions [19% in Asia (17), 11% in Latin America and the Caribbean (26), and 1% in Africa (24)]. Also similar to Europe and Australia and New Zealand (14, 27), HSV-1 detection in genital herpes increased with time. Such indicators, along with the large difference in seroprevalence between children and adults, are classic indicators defining a shift in HSV-1 epidemiology, from oral to increasingly genital acquisition, as observed in the United States and other Western countries (5, 7, 13, 14, 27). In context of the global evidence for the epidemiology of this infection, and based on pooling the different lines of evidence generated in this study, it appears that there is an ongoing HSV-1 epidemiological transition in Canada whereby HSV-1 infection plays an increasing role as a sexually transmitted infection.

These findings are consistent with findings of a study for HSV-2 infection in Canada that estimated HSV-2’s contribution to genital herpes at 62% and decreasing with time (22). Women were more affected by HSV-1 genital herpes than men, possibly reflecting an age gap in sexual partnerships, in which younger women partner with older men, or possibly reflecting a higher biological susceptibility of women who acquire the infection genitally (51, 52).

The present study has limitations. Included studies showed heterogeneity, yet most of the heterogeneity in seroprevalence and in proportion of HSV-1 detection in genital herpes reflected the natural heterogeneity that exists in HSV-1 epidemiology due to key epidemiological factors, such as age. More than 80% of the variation in seroprevalence and in proportion of HSV-1 detection in genital herpes was explained by few epidemiological factors through the meta-regression analyses (Tables 2, 4 and Supplementary Table S4).

While it is not known whether available measures are adequate to provide a representative sample of all studies that could theoretically be done in Canada during the study’s timeframe, there was a considerable number of studies from different parts of Canada, in different populations, and in different years to support that this number of studies may provide a random sample of studies that could theoretically have been done. Accordingly, the identified trends and patterns should be representative of the actual trends and patterns that exist in the overall population. Indeed, the identified trends and patterns in HSV-1 epidemiology are consistent with the trends and patterns observed in the United States (5, 7, 13), Western Europe (14), and Australia and New Zealand (27), as a consequence of a transition in the epidemiology of this infection in this part of the world (53). The overall consistency of HSV outcome measures in Canada with those found in other Western countries supports the validity of the inferences drawn in this study.

We estimated only an average overall trend for seroprevalence and proportion of HSV-1 detection in genital herpes, but these measures may have changed dramatically over decades, ebbing and flowing with changes to sexual practices, testing, and treatments, and influences of other infections such as the HIV epidemic (54). The number of included studies was not large enough to conduct more complex (or non-linear) regressions to assess different trends in different times.

In contrast to other regions (14, 17, 23, 26, 27), there was evidence of higher seroprevalence in Canada when the ELISA assay was employed, which may have led to a slight overestimation of the calculated pooled mean seroprevalence. It is worth noting that none of the identified studies were excluded based on diagnostic method-related problems associated with cross-reactivity with HSV-2 antibodies. Instead, exclusions were mainly due to inadequate information regarding the diagnostic assay used. Studies varied by sample size, sampling method, and response rate, yet there was no evidence that any of these factors affected the observed seroprevalence (Table 2 and Supplementary Table S4). On balance, while these limitations may affect some of the quantitative estimates in this study, they should not affect the overall findings of the study or their interpretation.

## Conclusions

Based on the totality of results presented in this study, HSV-1 epidemiology in Canada appears to be shifting toward less oral acquisition in childhood and more genital acquisition in adulthood. Two-thirds of youth are approaching sexual debut uninfected orally, and are at risk of being infected genitally, resulting in higher rates of genital herpes. Both HSV-1 seroprevalence and the proportion of HSV-1 detection in genital herpes appears to be increasing with time. These results emphasize the importance of research and surveillance to monitor HSV-1 seroprevalence and etiology of GUD and genital herpes, as well as the need for an HSV-1 vaccine to protect against acquisition of the infection. There is also a need to conduct mathematical modeling studies to quantitatively characterize HSV-1 transitioning epidemiology and to estimate its epidemiologic indicators such as incidence, past, present, and future, just as was done recently for the United States (5).

## Data availability statement

All relevant data are presented in the manuscript and its Supplementary material file. The dataset including the stratified HSV-1 seroprevalence measures and the stratified proportions of HSV-1 detection in genital herpes is posted at https://github.com/Abu-Raddad/HSV-1-in-Canada.git.

## Author contributions

SM, UF, MH, and LA conducted the systematic search, data extraction, and data analysis. SM wrote the first draft of the manuscript with LJA. LJA conceived the study and led the data extraction and analyses and interpretation of the results. All authors contributed to drafting and revising the manuscript.

## Funding

This work was supported by the Qatar National Research Fund (NPRP 9-040-3-008) and by pilot funding from the Biomedical Research Program at Weill Cornell Medicine in Qatar.

## Conflict of interest

The authors declare that the research was conducted in the absence of any commercial or financial relationships that could be construed as a potential conflict of interest.

## Publisher’s note

All claims expressed in this article are solely those of the authors and do not necessarily represent those of their affiliated organizations, or those of the publisher, the editors and the reviewers. Any product that may be evaluated in this article, or claim that may be made by its manufacturer, is not guaranteed or endorsed by the publisher.
