# Supplementary material for: Epidemiology of herpes simplex virus type 1 in Canada: systematic review, meta-analyses, and meta-regressions
Source: Front Public Health. 2023 Jul 14;11:1118249. doi: 10.3389/fpubh.2023.1118249 (PMC10375289; doi:10.3389/fpubh.2023.1118249)
Supplement: Supplementary file 1 [file Table_1.docx]

**Supplementary Material**

**Epidemiology of herpes simplex virus type 1 in Canada: Systematic review, meta-analyses, and meta-regressions**

**Table of Contents**

[**Table S1. Preferred Reporting Items for Systematic Reviews and Meta-analyses (PRISMA) checklist (1).** 3](#_Toc133953012)

[**Table S2. Data sources and search criteria for systematically reviewing HSV-1 epidemiology in Canada.** 6](#_Toc133953013)

[**Table S3. Studies reporting HSV-1 seroprevalence among different populations in Canada^*^.** 7](#_Toc133953014)

[**Figure S1. Forest plots of the pooled mean HSV-1 seroprevalence among different populations in Canada^*^.** 9](#_Toc133953015)

[**Table S4. Univariable and multivariable meta-regression analyses for HSV-1 seroprevalence in Canada with time trend assessed through a categorical term.** 10](#_Toc133953016)

[**Table S5. Studies reporting proportions of HSV-1 detection in clinically diagnosed genital ulcer disease and in laboratory-confirmed genital herpes in Canada^*^.** 11](#_Toc133953017)

[**Figure S2. Forest plot for the pooled mean proportion of HSV-1 detection in laboratory-confirmed genital herpes in Canada^*^.** 12](#_Toc133953018)

[**Table S6. Summary of the precision assessment and risk of bias assessment for the studies reporting HSV-1 seroprevalence in Canada.** 13](#_Toc133953019)

[**Table S7: Excluded publications/reports and reasons for exclusion.** 14](#_Toc133953020)

[**References** 15](#_Toc133953021)

# **Table S1. Preferred Reporting Items for Systematic Reviews and Meta-analyses (PRISMA) checklist (1).**

| **Section and Topic** | **Item #** | **Checklist item** | Location where item is reported |
| --- | --- | --- | --- |
| **TITLE** | | |  |
| Title | 1 | Identify the report as a systematic review. | p. 1 |
| **ABSTRACT** | | |  |
| Abstract | 2 | See the PRISMA 2020 for Abstracts checklist. | p. 2 |
| **INTRODUCTION** | | |  |
| Rationale | 3 | Describe the rationale for the review in the context of existing knowledge. | p. 3 |
| Objectives | 4 | Provide an explicit statement of the objective(s) or question(s) the review addresses. | p. 3 |
| **METHODS** | | |  |
| Eligibility criteria | 5 | Specify the inclusion and exclusion criteria for the review and how studies were grouped for the syntheses. | p. 3-4; Box 1 |
| Information sources | 6 | Specify all databases, registers, websites, organisations, reference lists and other sources searched or consulted to identify studies. Specify the date when each source was last searched or consulted. | p. 3-4; Box 1 |
| Search strategy | 7 | Present the full search strategies for all databases, registers and websites, including any filters and limits used. | Table S2 |
| Selection process | 8 | Specify the methods used to decide whether a study met the inclusion criteria of the review, including how many reviewers screened each record and each report retrieved, whether they worked independently, and if applicable, details of automation tools used in the process. | p. 3-4; Box 1 |
| Data collection process | 9 | Specify the methods used to collect data from reports, including how many reviewers collected data from each report, whether they worked independently, any processes for obtaining or confirming data from study investigators, and if applicable, details of automation tools used in the process. | p. 4; Box 1 |
| Data items | 10a | List and define all outcomes for which data were sought. Specify whether all results that were compatible with each outcome domain in each study were sought (e.g. for all measures, time points, analyses), and if not, the methods used to decide which results to collect. | Box 1 |
|  | 10b | List and define all other variables for which data were sought (e.g. participant and intervention characteristics, funding sources). Describe any assumptions made about any missing or unclear information. | Box 1 |
| Study risk of bias assessment | 11 | Specify the methods used to assess risk of bias in the included studies, including details of the tool(s) used, how many reviewers assessed each study and whether they worked independently, and if applicable, details of automation tools used in the process. | p. 4; Box 1 |
| Effect measures | 12 | Specify for each outcome the effect measure(s) (e.g. risk ratio, mean difference) used in the synthesis or presentation of results. | p. 4; Box 1 |
| Synthesis methods | 13a | Describe the processes used to decide which studies were eligible for each synthesis (e.g. tabulating the study intervention characteristics and comparing against the planned groups for each synthesis (item #5)). | p. 4; Box 1 |
|  | 13b | Describe any methods required to prepare the data for presentation or synthesis, such as handling of missing summary statistics, or data conversions. | Box 1 |
|  | 13c | Describe any methods used to tabulate or visually display results of individual studies and syntheses. | Box 1 |
|  | 13d | Describe any methods used to synthesize results and provide a rationale for the choice(s). If meta-analysis was performed, describe the model(s), method(s) to identify the presence and extent of statistical heterogeneity, and software package(s) used. | p. 5; Box 1 |
|  | 13e | Describe any methods used to explore possible causes of heterogeneity among study results (e.g. subgroup analysis, meta-regression). | p. 5; Box 1 |
|  | 13f | Describe any sensitivity analyses conducted to assess robustness of the synthesized results. | p. 5 |
| Reporting bias assessment | 14 | Describe any methods used to assess risk of bias due to missing results in a synthesis (arising from reporting biases). | NA |
| Certainty assessment | 15 | Describe any methods used to assess certainty (or confidence) in the body of evidence for an outcome. | NA |
| **RESULTS** | | |  |
| Study selection | 16a | Describe the results of the search and selection process, from the number of records identified in the search to the number of studies included in the review, ideally using a flow diagram. | p. 4-5; Figure 1 |
|  | 16b | Cite studies that might appear to meet the inclusion criteria, but which were excluded, and explain why they were excluded. | Figure 1 |
| Study characteristics | 17 | Cite each included study and present its characteristics. | Tables S3 and S5 |
| Risk of bias in studies | 18 | Present assessments of risk of bias for each included study. | p. 6; Table S6 |
| Results of individual studies | 19 | For all outcomes, present, for each study: (a) summary statistics for each group (where appropriate) and (b) an effect estimate and its precision (e.g. confidence/credible interval), ideally using structured tables or plots. | p. 5-6; Tables 1 and 3; Figures S1 and S2; Tables S3 and S5 |
| Results of syntheses | 20a | For each synthesis, briefly summarise the characteristics and risk of bias among contributing studies. | Tables S3, S5, and S6 |
|  | 20b | Present results of all statistical syntheses conducted. If meta-analysis was done, present for each the summary estimate and its precision (e.g. confidence/credible interval) and measures of statistical heterogeneity. If comparing groups, describe the direction of the effect. | p. 5-6; Tables 1 and 3 |
|  | 20c | Present results of all investigations of possible causes of heterogeneity among study results. | p. 5-6; Tables 2 and 4; Table S4 |
|  | 20d | Present results of all sensitivity analyses conducted to assess the robustness of the synthesized results. | Table S4 |
| Reporting biases | 21 | Present assessments of risk of bias due to missing results (arising from reporting biases) for each synthesis assessed. | NA |
| Certainty of evidence | 22 | Present assessments of certainty (or confidence) in the body of evidence for each outcome assessed. | NA |
| **DISCUSSION** | | |  |
| Discussion | 23a | Provide a general interpretation of the results in the context of other evidence. | p. 7-8 |
|  | 23b | Discuss any limitations of the evidence included in the review. | p. 7-8 |
|  | 23c | Discuss any limitations of the review processes used. | p. 7-8 |
|  | 23d | Discuss implications of the results for practice, policy, and future research. | p. 8 |
| **OTHER INFORMATION** | | |  |
| Registration and protocol | 24a | Provide registration information for the review, including register name and registration number, or state that the review was not registered. | NA |
|  | 24b | Indicate where the review protocol can be accessed, or state that a protocol was not prepared. | NA |
|  | 24c | Describe and explain any amendments to information provided at registration or in the protocol. | NA |
| Support | 25 | Describe sources of financial or non-financial support for the review, and the role of the funders or sponsors in the review. | p. 9 |
| Competing interests | 26 | Declare any competing interests of review authors. | p. 9 |
| Availability of data, code and other materials | 27 | Report which of the following are publicly available and where they can be found: template data collection forms; data extracted from included studies; data used for all analyses; analytic code; any other materials used in the review. | p. 9 |

Abbreviations: NA, not applicable; p, page(s).

# **Table S2. Data sources and search criteria for systematically reviewing HSV-1** **epidemiology in Canada.**

| **PubMed (last searched December 6, 2021):** |
| --- |
| (Simplexvirus[MeSH] OR Herpes Simplex[MeSH] OR herpes genitalis[MESH] OR genital herpes[Text] OR Herpes Genitalis[Text] OR Stomatitis Herpetic[Text] OR Herpes Labialis[Text] OR Human herpes virus[Text] OR Herpes Hominis[Text] OR Herpes virus[Text] OR HSV type-1[Text] OR HSV type 1[Text] OR HSV1[Text] OR HSV-1[Text] OR HSV 1[Text] OR Herpes simplex virus type 1[Text] OR Herpes simplex virus type-1[Text] OR herpes simplex virus 1[Text] OR herpes simplex virus-1[Text] OR herpes simplex type 1[Text] OR herpes simplex type-1[Text] OR herpes simplex 1[Text] OR herpes simplex-1[Text] OR Herpesvirus type 1[Text] OR Herpesvirus type-1[Text] OR Herpesvirus 1[Text] OR Herpesvirus-1[Text] OR Herpes virus type 1[Text] OR Herpes virus type-1[Text] OR Herpes virus 1[Text] OR Herpes virus-1[Text] OR HSV type-2[Text] OR HSV type 2[Text] OR HSV2[Text] OR HSV-2[Text] OR HSV [Text] OR Herpes simplex virus type 2[Text] OR Herpes simplex virus type-2[Text] OR herpes simplex virus 2[Text] OR herpes simplex virus-2[Text] OR herpes simplex type 2[Text] OR herpes simplex type-2[Text] OR herpes simplex 2[Text] OR herpes simplex-2[Text] OR Herpesvirus type 2[Text] OR Herpesvirus type-2[Text] OR Herpesvirus 2[Text] OR Herpesvirus-2[Text] OR Herpes virus type 2[Text] OR Herpes virus type-2[Text] OR Herpes virus-2[Text]) AND (Canada[MeSH]) OR (Canad*[Text]) |
| **Embase (last searched December 6, 2021):** |
| (exp Herpes simplex/ or exp Simplexvirus/ or exp herpes simplex virus/ or exp genital herpes/) OR (Herpes simplex or Herpes simplex virus or human herpes virus or genital herpes or Herpes Genitalis or herpes labialis or herpetic stomatitis).mp. OR (HSV type-1 or HSV type 1 or HSV1 or HSV-1 or Herpes simplex virus type 1 or Herpes simplex virus type-1 or herpes simplex virus 1 or herpes simplex virus-1 or herpes simplex type 1 or herpes simplex type-1 or herpes simplex 1 or herpes simplex-1 or Herpesvirus type 1 or Herpesvirus type-1 or Herpesvirus 1 or Herpesvirus-1 or Herpes virus type 1 or Herpes virus type-1 or Herpes virus 1 or Herpes virus-1).mp. OR (HSV type-2 or HSV type 2 or HSV2 or HSV-2 or HSV 2 or Herpes simplex virus type 2 or Herpes simplex virus type-2 or herpes simplex virus 2 or herpes simplex virus-2 or herpes simplex type 2 or herpes simplex type-2 or herpes simplex 2 or herpes simplex-2 or Herpesvirus type 2 or Herpesvirus type-2 or Herpesvirus 2 or Herpesvirus-2 or Herpes virus type 2 or Herpes virus type-2 or Herpes virus 2 or Herpes virus-2).mp. AND (exp Canada/) OR (Canad*).mp. |
| **Institutional websites of public health authorities in Canada (last searched April 25, 2023):** |
| 1. Public Health Agency of Canada: <https://www.canada.ca/en/public-health.html> 2. HealthLink BC: <https://www.healthlinkbc.ca/> 3. Saskatchewan Health: <https://www.saskatchewan.ca/> 4. Canadian Federation for Sexual Health: <https://www.actioncanadashr.org/about/who-we-are> 5. Santé de Services Sociaux Québec: <https://www.msss.gouv.qc.ca/> 6. Government of Prince Edward Island: <https://www.princeedwardisland.ca/en/topic/health-pei> |

Abbreviations: HSV-1, herpes simplex virus type 1; HSV-2, herpes simplex virus type.

# **Table S3. Studies reporting HSV-1 seroprevalence among different populations in Canada^*^.**

| **Author, year** | **Year(s) of data collection** | **City, province** | | **Study site** | **Study design** | **Sampling methodology** | **HSV-1 serological assay** | **Population** | **Sample size** | **HSV-1 seroprevalence (%)** |
| --- | --- | --- | --- | --- | --- | --- | --- | --- | --- | --- |
| **General populations** | |  | |  |  |  |  |  |  |  |
| Garland, 1995 (2) | 1985-86 | Vancouver, BC | | Outpatient clinic | CS | RS | WB | Pregnant women | 426 | 54.2 |
| Gorfinkel, 2013 (3) | 2005-07 | Quebec, Alberta, Nova Scotia, BC, Manitoba, and Ontario | | Outpatient clinic | CS | Conv | WB | Healthy women with no history of herpes infection | 2,694 | 44.7 |
| Howard, 2003 (4) | 2000-01 | Ontario | | Outpatient clinic | CS | CRS | EIA | Healthy men | 979 | 48.1 |
| Howard, 2003 (4) | 2000-01 | Ontario | | Outpatient clinic | CS | CRS | EIA | Healthy women not under prenatal care | 638 | 63.0 |
| Howard, 2003 (4) | 2000-01 | Ontario | | Outpatient clinic | CS | CRS | EIA | Healthy women under prenatal care | 701 | 66.8 |
| McDonald, 1974a (5) | - | Montreal, Quebec | | Community | CC | Conv | Neutralization | Women without invasive carcinoma | 57 | 52.5 |
| McDonald, 1974a (5) | - | Montreal, Quebec | | Community | CC | Conv | Neutralization | Women without carcinoma in the situ | 50 | 46.0 |
| McDonald, 1974a (5) | - | Montreal, Quebec | | Community | CC | Conv | Neutralization | Women without cervical dysplasia | 39 | 41.0 |
| McDonald, 1974b (6) | 1968-71 | Montreal, Quebec | | Community | CS | RS | Neutralization | Women with income ≤ $3,500 | 148 | 44.9 |
| McDonald, 1974b (6) | 1968-71 | Montreal, Quebec | | Community | CS | RS | Neutralization | Women with income between $3,500-3,999 | 144 | 35.9 |
| McDonald, 1974b (6) | 1968-71 | Montreal, Quebec | | Community | CS | RS | Neutralization | Women with income between $4,000-4,799 | 157 | 38.1 |
| McDonald, 1974b (6) | 1968-71 | Montreal, Quebec | | Community | CS | RS | Neutralization | Women with income ≥ $4,800 | 115 | 28.2 |
| McDonald, 1974c (7) | - | Montreal, Quebec | | Community | CC | Conv | Neutralization | Women who had a child with a neural-tube defect | 125 | 46.4 |
| McDonald, 1974c (7) | - | Montreal, Quebec | | Community | CC | Conv | Neutralization | Women who had a child without a neural-tube defect | 125 | 54.4 |
| Patrick, 2001 (8) | 1999 | BC | | Outpatient clinic | CS | SRS | ELISA | Pregnant women | 1,215 | 58.9 |
| Remis, 2013 (9) | 2009-10 | Toronto, Ontario | | Outpatient clinic | CS | Conv | ELISA | Women from Africa and the Caribbean living in Toronto | 290 | 87.6 |
| **Clinical populations** | |  | |  |  |  |  |  |  |  |
| Garland, 1995 (2) | 1985-87 | Vancouver, BC | | Outpatient clinic | CS | Conv | WB | Pregnant women at high risk for genital herpes | 195 | 35.4 |
| Mak, 2011 (10) | - | BC | | Outpatient clinic | CS | Conv | EIA | Patients’ specimens from BCCDC | 1,556 | 75.1 |
| McDonald, 1974a (5) | - | Montreal, Quebec | | Community | CC | Conv | Neutralization | Women with invasive carcinoma | 57 | 47.4 |
| McDonald, 1974a (5) | - | Montreal, Quebec | | Community | CC | Conv | Neutralization | Women with carcinoma in the situ | 50 | 34.0 |
| McDonald, 1974a (5) | - | Montreal, Quebec | | Community | CC | Conv | Neutralization | Women with cervical dysplasia | 39 | 46.2 |
| Singh, 2005 (11) | 1994-95 | Alberta | | Outpatient clinic | CS | Conv | ELISA | Patients attending an STD clinic | 6,555 | 55.7 |
| Zahariadis, 2010 (12) | 2007 | Alberta | | Outpatient clinic | CS | Conv | WB | Samples from University of Alberta Hospital | 189 | 66.1 |
| Zahariadis, 2010 (12) | 2004-06 | Manitoba | | Outpatient clinic | CS | Conv | WB | Samples from the National Microbiology Laboratory | 155 | 65.8 |
| **Other populations** |  |  | |  |  |  |  |  |  |  |
| HIV positive populations | | |  | |  |  |  |  |  |  |
| Remis, 2013 (9) | 2009-10 | Toronto, Ontario | | Outpatient clinic | CS | Conv | ELISA | HIV-positive women | 123 | 90.2 |
| Remis, 2016 (13) | 2010-12 | Toronto, Ontario | | Outpatient clinic | CS | Conv | ELISA | MSM who are HIV-positive | 288 | 78.5 |
| Romanowski, 2009 (14) | 2005-07 | Edmonton, Alberta; Quebec; Toronto, Ontario | | Outpatient clinic | CS | Conv | ELISA | HIV-positive patients | 629 | 78.1 |
| Tan, 2013 (15) | 2006-11 | Toronto and Hamilton, Ontario | | Outpatient clinic | Cohort^†^ | Conv | ELISA | HIV-positive patients | 218 | 73.9 |
| Yi, 2013 (16) | - | Toronto, Ontario | | Outpatient clinic | RCT^†^ | Conv | ELISA | HIV-positive patients and receiving placebo | 20 | 75.0 |
| Yi, 2013 (16) | - | Toronto, Ontario | | Outpatient clinic | RCT^†^ | Conv | ELISA | HIV-positive patients and receiving valacyclovir 500 mg | 19 | 73.7 |
| Yi, 2013 (16) | - | Toronto, Ontario | | Outpatient clinic | RCT^†^ | Conv | ELISA | HIV-positive patients and receiving valacyclovir 1 g | 20 | 85.0 |
| Men who have sex with men | | |  | |  |  |  |  |  |  |
| Remis, 2016 (13) | 2010-12 | Toronto, Ontario | | Outpatient clinic | CS | Conv | ELISA | MSM who are HIV-negative | 144 | 69.4 |

^*^The included measures are the overall (not stratified) HSV-1 seroprevalence measures extracted from each of these studies.

^†^The reported study design is the original study design (cross-sectional, cohort, or randomized controlled trial). The included seroprevalence measures are those for the baseline measures at the beginning of the study.

Abbreviations: BC, British Columbia; BCCDC, British Columbia Centre for Disease Control; CC, case-control; Conv, convenience; CRS, cluster random sampling; CS, cross-sectional; EIA, enzyme immunoassay; ELISA, enzyme-linked immunosorbent type-specific assay; HIV, human immunodeficiency virus; HSV-1, herpes simplex virus type 1; MSM, men who have sex with men; RCT, randomized controlled trial; RS, random sampling; SRS, stratified random sampling; STD, sexually transmitted disease; WB, western blot.

# **Figure S1. Forest plots of the pooled mean HSV-1 seroprevalence among different populations in Canada^*^.**





^*^Each line represents an HSV-1 seroprevalence measure in a specific stratum.

# **Table S4. Univariable and multivariable meta-regression analyses for HSV-1 seroprevalence in Canada with time trend assessed through a categorical term.**

|  | | | **Outcome measures** | **Samples** | | **Univariable analysis^*^** | | | | | | | | **Multivariable analyses^*†^** | | | | | |
| --- | --- | --- | --- | --- | --- | --- | --- | --- | --- | --- | --- | --- | --- | --- | --- | --- | --- | --- | --- |
|  |  |  |  |  |  |  |  |  |  |  |  |  |  | **Model 3^‡^** | | | **Model 4^§^** | | |
|  | | | **Total n** | **Total N** | | ***RR* (95% CI)** | | **p-value** | | **LR test p-value** | | **Adjusted R^2^ (%)** | | ***aRR* (95% CI)** | **p-value** | | ***aRR* (95% CI)** | **p-value** | |
| **Population Characteristics** | **Age bracket** | Children | 4 | | 134 | | 1.00 | | - | | <0.001 | | 14.68 | 1.00 | | - | - | | - |
|  |  | Adults | 75 | | 18,026 | | 2.68 (1.68-4.29) | | <0.001 | |  | |  | 2.17 (1.38-3.41) | | 0.001 | - | | - |
|  | **Age group** | <20 | 15 | | 2,677 | | 1.00 | | - | | <0.001 | | 36.72 | - | | - | 1.00 | | - |
|  |  | 20-29 | 13 | | 2,519 | | 1.40 (1.13-1.73) | | 0.003 | |  | |  | - | | - | 1.29 (1.12-1.48) | | <0.001 |
|  |  | 30-39 | 8 | | 783 | | 1.76 (1.38-2.24) | | <0.001 | |  | |  | - | | - | 1.59 (1.36-1.86) | | <0.001 |
|  |  | ≥40 | 7 | | 441 | | 1.94 (1.50-2.51) | | <0.001 | |  | |  | - | | - | 1.68 (1.38-2.03) | | <0.001 |
|  |  | Mixed | 36 | | 11,740 | | 1.46 (1.21-1.75) | | <0.001 | |  | |  | - | | - | 1.82 (1.50-2.22) | | <0.001 |
|  | **Sex** | Women | 59 | | 7,388 | | 1.00 | | - | | 0.006 | | 14.97 | 1.00 | | - | 1.00 | | - |
|  |  | Men | 11 | | 1,411 | | 0.92 (0.75-1.13) | | 0.404 | |  | |  | 0.73 (0.61-0.88) | | 0.001 | 0.74 (0.65-0.84) | | <0.001 |
|  |  | Mixed | 9 | | 9,361 | | 1.40 (1.12-1.74) | | 0.003 | |  | |  | 1.10 (0.82-1.48) | | 0.526 | 1.01 (0.82-1.23) | | 0.960 |
|  | **Population type** | Healthy | 63 | | 7,903 | | 1.00 | | - | | <0.001 | | 22.91 | 1.00 | | - | 1.00 | | - |
|  |  | Clinical | 8 | | 8,796 | | 1.06 (0.85-1.33) | | 0.589 | |  | |  | 0.95 (0.72-1.27) | | 0.742 | 0.83 (0.67-1.04) | | 0.100 |
|  |  | Other | 8 | | 1,461 | | 1.56 (1.25-1.95) | | <0.001 | |  | |  | 1.33 (0.97-1.83) | | 0.078 | 1.03 (0.81-1.32) | | 0.779 |
| **Study methodology characteristics** | **Assay type** | Western blot | 7 | | 3,659 | | 1.00 | | - | | <0.001 | | 16.88 | 1.00 | | - | 1.00 | | - |
|  |  | ELISA | 44 | | 13,395 | | 1.16 (0.92-1.46) | | 0.217 | |  | |  | 1.24 (0.98-1.58) | | 0.068 | 1.26 (1.07-1.47) | | 0.005 |
|  |  | Neutralization | 28 | | 1,106 | | 0.83 (0.64-1.07) | | 0.146 | |  | |  | 0.99 (0.75-1.32) | | 0.976 | 0.84 (0.68-1.04) | | 0.109 |
|  | **Sample size^¶^** | <100 | 3 | | 59 | | 1.00 | | - | | 0.039 | | 5.48 | 1.00 | | - | 1.00 | | - |
|  |  | ≥100 | 76 | | 18,101 | | 0.66 (0.45-0.98) | | 0.039 | |  | |  | 1.21 (0.81-1.79) | | 0.343 | 1.06 (0.73-1.55) | | 0.741 |
|  | **Sampling method** | Probability based | 54 | | 4,523 | | 1.00 | | - | | 0.020 | | 5.72 | 1.00 | | - | 1.00 | | - |
|  |  | Non-probability based | 25 | | 13,637 | | 1.21 (1.03-1.42) | | 0.020 | |  | |  | 1.08 (0.85-1.38) | | 0.530 | 1.04 (0.88-1.22) | | 0.679 |
|  | **Response rate** | <80% | 21 | | 7,119 | | 1.00 | | - | | 0.001 | | 8.21 | 1.00 | | - | 1.00 | | - |
|  |  | Unclear | 58 | | 11,041 | | 1.38 (1.14-1.66) | | 0.001 | |  | |  | 1.01 (0.78-1.32) | | 0.922 | 1.08 (0.90-1.31) | | 0.394 |
| **Year of data collection category** | | ≤2000 | 37 | | 10,097 | | 1.00 | | - | | 0.008 | | 6.03 | 1.00 | | - | 1.00 | | - |
|  |  | >2000 | 42 | | 8,063 | | 1.23 (1.06-1.43) | | 0.008 | |  | |  | 1.07 (0.91-1.26) | | 0.400 | 1.16 (1.04-1.30) | | 0.009 |

^*^The meta-regression analyses were based on the 79 stratified HSV-1 seroprevalence.

^†^Two multivariable models were conducted, one including age bracket (children *versus* adults) and one including age group.

^‡^Variance explained by the final multivariable model 3 (adjusted *R^2^*) = 48.44%.

^§^Variance explained by the final multivariable model 4 (adjusted *R^2^*) = 81.40%.

^¶^Sample size denotes the sample size of each study population found in the original publication.

Abbreviations: *aRR*, adjusted risk ratio; CI, confidence interval; ELISA, enzyme-linked immunosorbent type-specific assay; HSV-1, herpes simplex virus type 1; *RR*, risk ratio.

# **Table S5. Studies reporting proportions of HSV-1 detection in clinically diagnosed genital ulcer disease and in laboratory-confirmed genital herpes in Canada^*^.**

| **Author, year** | **Year(s) of data collection** | **City, province** | **Study site** | **Study design** | **Sampling methodology** | **HSV-1 biological assay** | **Population** | **Sample size** | **Proportion of HSV-1 detection (%)** |
| --- | --- | --- | --- | --- | --- | --- | --- | --- | --- |
| **Patients with clinically diagnosed genital ulcer disease** | | | | | | | | | |
| Mak, 2011 (17) | 2010 | Not reported | Outpatient clinic | CS | Conv | PCR | Specimens from genital lesions | 1,294 | 41.8 |
| Wong, 2016 (18) | 2012-14 | Alberta | Outpatient clinic | CS | Conv | PCR | Patients with genital lesions | 6,836 | 20.8 |
| **Patients with clinically diagnosed genital herpes** | | | | | | | | | |
| Forward, 2003 (19) | 1998-01 | Nova Scotia | Outpatient clinic | CS | Conv | IF | Genital cultures from women | 1,790 | 58.2 |
| Forward, 2003 (19) | 1998-01 | Nova Scotia | Outpatient clinic | CS | Conv | IF | Genital cultures from men | 468 | 36.8 |
| Garceau, 2012 (20) | 2006-08 | New Brunswick | Outpatient clinic | CS | Conv | Mab | Patients with genital lesions | 764 | 62.6 |
| Gilbert, 2011 (21) | 1997-05 | BC | Outpatient clinic | Cohort^†^ | Conv | Mab | Patients with genital herpes | 27,389 | 37.5 |
| Mak, 2011 (17) | 2010 | Not reported | Outpatient clinic | CS | Conv | PCR | Specimens from genital lesions | 1,259 | 43.0 |
| Sacks, 1991 (22) | - | Vancouver, BC; Toronto, Ontario; and Edmonton, Alberta | Outpatient clinic | RCT^†^ | Conv | Mab | Patients with genital herpes | 200 | 1.0 |
| Shabi, 2019 (23) | - | Nova Scotia | Outpatient clinic | CS | Conv | PCR | Anogenital specimens taken from patients | 32 | 40.6 |
| Wong, 2016 (18) | 2012-14 | Alberta | Outpatient clinic | CS | Conv | PCR | Patients with genital lesions | 3,001 | 47.4 |

^*^The included measures are the overall (not stratified) proportions of HSV-1 detection in clinically diagnosed genital ulcer disease and in laboratory-confirmed genital herpes.

^†^The reported study design is the original study design (cross-sectional, cohort, or randomized controlled trial). The included measures for the proportion of HSV-1 detection are those for the baseline measures at the beginning of the study.

Abbreviations: BC, British Columbia; Conv, convenience; CS, cross sectional; HSV-1, herpes simplex virus type 1; IF, immunofluorescence; Mab, monoclonal antibody; MSM, men who have sex with men; PCR, polymerase chain reaction; RCT, randomized controlled trial.

# **Figure S2. Forest plot for the pooled mean proportion of HSV-1 detection in laboratory-confirmed genital herpes in Canada****^*^.**





^*^Each line represents a proportion measure of HSV-1 detection in genital herpes in a specific stratum.

# **Table S6. Summary of the precision assessment and risk of bias assessment for the studies reporting HSV-1 seroprevalence in Canada.**

| **Quality assessment** | **HSV-1 seroprevalence measures** | |
| --- | --- | --- |
|  | **Number of studies** | **%** |
| **Precision of seroprevalence measures^*^** | | |
| Low precision | 3 | 9.4 |
| High precision | 29 | 90.6 |
| **Risk of bias quality domain^†^** | | |
| **Sampling methodology** | | |
| Low risk of bias | 9 | 28.1 |
| High risk of bias | 23 | 71.9 |
| **Response rate** | | |
| Low risk of bias | 0 | 0 |
| High risk of bias | 5 | 15.6 |
| Unclear risk of bias | 27 | 84.4 |
| **Summary of the risk of bias assessment** | | |
| **Low risk of bias** |  |  |
| In at least one quality domain | 9 | 28.1 |
| In both quality domains | 0 | 0 |
| **High risk of bias** |  |  |
| In at least one quality domain | 27 | 84.4 |
| In both quality domains | 1 | 3.1 |
| **Seroprevalence studies where risk of bias assessment was possible** | **32** | **100.0** |

^*^Precision was assessed based on the overall sample size (not each stratum subsample size) of the study as reported in the publication.

^†^Risk of bias was assessed based on the overall sample size (not each stratum subsample size) of the study as reported in the publication.

Abbreviations: HSV-1, herpes simplex virus type 1.

# **Table S7: Excluded publications/reports and reasons for exclusion.**

| **Reference** | **Reason for exclusion** |
| --- | --- |
| **Publications identified from databases** | |
| Alary 1989, Allen 2013, Almeida 2010, Anema 2011, Anonymous 1986a, Anonymous 1986b, Anonymous 1986c, Anonymous 1993, Auger 2018, Bodsworth 2008, Delva 1984, Diaz-Mitoma 1998, Eisler 2010, Embil 1985, Embil 1975, Ernst 2021, Ezzat 2012, Gesink 2018, Gill 1988, Guibinga 1996, Gully 1995, Hamandi 2016, Hammerberg 1983, Hankins 1994, Hockin 1985, Jassem 2016, Kariyawasam 2016, Kaspirkova 2016, Kolski 1998, Logie 2015, McClymont 2019, McSherry 1986, Nicolle 1986, Piret 2001, Plotnikoff 2020, Rawls 1986, Redmond 1990, Shukalek 2021, Tremblay 1997, Tremblay 2015, Vynograd 2000, Wilson 1995, Zuckerman 2013 (24-66) | Full-text did not include data on relevant indicators. |
| Kaul 2010, Liu 2014, Shannon 2014, Tan 2011, Tan 2014, Tan 2015a, Tan 2015b (67-73) | Duplicate data. |
| Jessamine 1985 (74) | Insufficient information on the diagnostic methods to assess the validity of the assays and specimen type was not explicitly specified for genital herpes measure. |
| **Reports identified from institutional websites of Canadian public health authorities** | |
| Government of Prince Edward Island 2014, Public Health Agency of Canada 2007 (75, 76) | Full-text did not include original data on relevant indicators. |
| Li 2008 (77) | Insufficient information on the diagnostic methods to assess the validity of the assays and specimen type was not explicitly specified for genital herpes measure. |

# **References**

1. Page MJ, McKenzie JE, Bossuyt PM, Boutron I, Hoffmann TC, Mulrow CD, Shamseer L, Tetzlaff JM, Akl EA, Brennan SE, Chou R, Glanville J, Grimshaw JM, Hróbjartsson A, Lalu MM, Li T, Loder EW, Mayo-Wilson E, McDonald S, McGuinness LA, Stewart LA, Thomas J, Tricco AC, Welch VA, Whiting P, Moher D. The PRISMA 2020 statement: an updated guideline for reporting systematic reviews. Bmj. 2021 Mar 29;372:n71. eng. Epub 2021/03/31. doi:10.1136/bmj.n71. Cited in: Pubmed; PMID 33782057.

2. Garland SM, Lee TN, Ashley RL, Corey L, Sacks SL. Automated microneutralization: method and comparison with western blot for type-specific detection of herpes simplex antibodies in two pregnant populations. J Virol Methods. 1995 Nov;55(3):285-94. eng. Epub 1995/11/01. doi:10.1016/0166-0934(95)00061-9. Cited in: Pubmed; PMID 8609194.

3. Gorfinkel IS, Aoki F, McNeil S, Dionne M, Shafran SD, Zickler P, Halperin S, Langley J, Bellamy A, Schulte J, Heineman T, Belshe R. Seroprevalence of HSV-1 and HSV-2 antibodies in Canadian women screened for enrolment in a herpes simplex virus vaccine trial. International Journal of STD and AIDS. 2013 November;24(5):345-349. Cited in: Pubmed; PMID 370229562.

4. Howard M, Sellors JW, Jang D, Robinson NJ, Fearon M, Kaczorowski J, Chernesky M. Regional distribution of antibodies to herpes simplex virus type 1 (HSV-1) and HSV-2 in men and women in Ontario, Canada. Journal of Clinical Microbiology. 2003 01 Jan;41(1):84-89. Cited in: Pubmed; PMID 36098558.

5. McDonald AD, Williams MC, Manfreda J, West R. Neutralizing antibodies to herpesvirus types 1 and 2 in carcinoma of the cervix, carcinoma in situ and cervical dysplasia. Am J Epidemiol. 1974a Aug;100(2):130-5. eng. Epub 1974/08/01. doi:10.1093/oxfordjournals.aje.a112015. Cited in: Pubmed; PMID 4368955.

6. McDonald AD, Williams MC, West R, Stewart J. Neutralizing antibodies to herpesvirus types 1 and 2 in Montreal women. Am J Epidemiol. 1974b Aug;100(2):124-9. eng. Epub 1974/08/01. doi:10.1093/oxfordjournals.aje.a112014. Cited in: Pubmed; PMID 4368956.

7. McDonald AD, Williams MC, West R. Neural-tube defects and herpesvirus type 2--test of a hypothesis. Teratology. 1974 Aug;10(1):13-6. eng. Epub 1974/08/01. doi:10.1002/tera.1420100104. Cited in: Pubmed; PMID 4368952.

8. Patrick DM, Dawar M, Cook DA, Krajden M, Ng HC, Rekart ML. Antenatal seroprevalence of herpes simplex virus type 2 (HSV-2) in canadian women: HSV-2 prevalence increases throughout the reproductive years. Sexually Transmitted Diseases. 2001;28(7):424-428. Cited in: Pubmed; PMID 32613687.

9. Remis RS, Liu J, Loutfy M, Tharao W, Rebbapragada A, Perusini SJ, Chieza L, Saunders M, Green-Walker L, Kaul R. The epidemiology of sexually transmitted co-infections in HIV-positive and HIV-negative African-Caribbean women in Toronto. BMC Infect Dis. 2013 Nov 17;13:550. eng. Epub 2013/11/19. doi:10.1186/1471-2334-13-550. Cited in: Pubmed; PMID 24238493.

10. Mak A, Petric M, Krajden M, Severini A. Evaluation of the focus diagnostic herpeselect 1 and 2 elisa IGG [Conference Abstract]. Canadian Journal of Infectious Diseases and Medical Microbiology. 2011 Spring;22;SA:37A.

11. Singh AE, Romanowski B, Wong T, Gourishankar S, Myziuk L, Fenton J, Preiksaitis JK. Herpes simplex virus seroprevalence and risk factors in 2 Canadian sexually transmitted disease clinics. Sex Transm Dis. 2005 Feb;32(2):95-100. eng. Epub 2005/01/26. doi:10.1097/01.olq.0000151415.78210.85. Cited in: Pubmed; PMID 15668615.

12. Zahariadis G, Severini A. Evaluation of a novel serology algorithm to detect herpes simplex virus 1 or 2 antibodies. Sex Transm Dis. 2010 Nov;37(11):696-9. eng. Epub 2010/08/10. doi:10.1097/OLQ.0b013e3181e2cdab. Cited in: Pubmed; PMID 20693937.

13. Remis RS, Liu J, Loutfy MR, Tharao W, Rebbapragada A, Huibner S, Kesler M, Halpenny R, Grennan T, Brunetta J, Smith G, Reko T, Kaul R. Prevalence of Sexually Transmitted Viral and Bacterial Infections in HIV-Positive and HIV-Negative Men Who Have Sex with Men in Toronto. PLoS One. 2016;11(7):e0158090. eng. Epub 2016/07/09. doi:10.1371/journal.pone.0158090. Cited in: Pubmed; PMID 27391265.

14. Romanowski B, Myziuk LN, Walmsley SL, Trottier S, Singh AE, Houston S, Joffe M, Chiu I. Seroprevalence and risk factors for herpes simplex virus infection in a population of HIV-infected patients in Canada. Sex Transm Dis. 2009 Mar;36(3):165-9. eng. Epub 2008/12/23. doi:10.1097/OLQ.0b013e31818d3fb6. Cited in: Pubmed; PMID 19098690.

15. Tan DH, Raboud JM, Kaul R, Brunetta J, Kaushic C, Kovacs C, Lee E, Luetkehoelter J, Rachlis A, Smaill F, Smieja M, Walmsley SL. Herpes simplex virus type 2 coinfection does not accelerate CD4 count decline in untreated HIV infection. Clin Infect Dis. 2013 Aug;57(3):448-57. eng. Epub 2013/04/11. doi:10.1093/cid/cit208. Cited in: Pubmed; PMID 23572481.

16. Yi TJ, Walmsley S, Szadkowski L, Raboud J, Kaul R, Rajwans N, Kain KC, Shannon B, Kumar S, Tan DH. A pilot randomized controlled trial of valacyclovir for attenuating inflammation and immune activation in HIV, HSV-2 co-infected adults on suppressive antiretroviral therapy. Canadian Journal of Infectious Diseases and Medical Microbiology. 2013 Spring;SA):29A. Cited in: Pubmed; PMID 71971677.

17. Mak A, Petric M, Krajden M, Tang P. Detection of varicella-zoster virus (VZV) from genital specimens. Canadian Journal of Infectious Diseases and Medical Microbiology. 2011 Spring;SA):12A. Cited in: Pubmed; PMID 70476166.

18. Wong AA, Pabbaraju K, Wong S, Tellier R. Development of a multiplex real-time PCR for the simultaneous detection of herpes simplex and varicella zoster viruses in cerebrospinal fluid and lesion swab specimens. J Virol Methods. 2016 Mar;229:16-23. eng. Epub 2015/12/30. doi:10.1016/j.jviromet.2015.12.009. Cited in: Pubmed; PMID 26711555.

19. Forward KR, Lee SHS. Predominance of herpes simplex virus type 1 from patients with genital herpes in Nova Scotia. Canadian Journal of Infectious Diseases. 2003 March/April;14(2):94-96.

20. Garceau R, Leblanc D, Thibault L, Girouard G, Mallet M. Herpes simplex virus type 1 is the leading cause of genital herpes in New Brunswick. Canadian Journal of Infectious Diseases and Medical Microbiology. 2012 Spring;23(1):15-18.

21. Gilbert M, Li X, Petric M, Krajden M, Isaac-Renton JL, Ogilvie G, Rekart ML. Using centralized laboratory data to monitor trends in Herpes Simplex Virus type 1 and 2 infection in British Columbia and the changing Etiology of genital Herpes. Canadian Journal of Public Health. 2011 May-June;102(3):225-229. Cited in: Pubmed; PMID 362224106.

22. Sacks SL, Tyrrell LD, Lawee D, Schlech IW, Gill MJ, Aoki FY, Martel AY, Singer J. Randomized, double-blind, placebo-controlled, clinic-initiated, canadian multicenter trial of topical edoxudine 3.0% cream in the treatment of recurrent genital herpes. Journal of Infectious Diseases. 1991;164(4):665-672.

23. Shabi Y, Jackson C, Sarty D, Heinstein C, MacDonald J, Ng J, Mazzulli T, Hatchette TF, LeBlanc JJ. Comparison of two automated methods for detection and differentiation of herpes simplex virus in clinical specimens. J Clin Virol. 2019 Aug;117:85-88. eng. Epub 2019/07/01. doi:10.1016/j.jcv.2019.04.010. Cited in: Pubmed; PMID 31255793.

24. Alary M, Joly JR, Poulin C. Incidence of four sexually transmitted diseases in a rural community: a prospective study. Am J Epidemiol. 1989 Sep;130(3):547-56. eng. Epub 1989/09/01. doi:10.1093/oxfordjournals.aje.a115369. Cited in: Pubmed; PMID 2763999.

25. Allen UD. Minimizing infection risks after paediatric organ transplants: Advice for practitioners. Paediatrics and Child Health (Canada). 2013 March;18(3):143-148. Cited in: Pubmed; PMID 368458853.

26. Almeida S, Filipe A, Neves R, Spinola ACF, Tanguay M, Ortuno J, Farre A, Torns A. Mycophenolate mofetil 500-mg tablet under fasting conditions: Single-dose, randomized-sequence, open-label, four-way replicate crossover, bioequivalence study in healthy subjects. Clinical Therapeutics. 2010 March;32(3):556-574. Cited in: Pubmed; PMID 358767393.

27. Anema A, Raboud J, Su D, Zakaryan A, Klein MB, Swan T, Palmer A, Machouf N, Rourke S, Loutfy M, Hosein S, Tsoukas C, Hogg RS, Cooper C. Relationship of chronic hepatitis C infection to rates of AIDS defining illnesses in a Canadian cohort of HIV seropositive individuals receiving highly active antiretroviral therapy. Canadian Journal of Infectious Diseases and Medical Microbiology. 2011 Spring;SB):60B. Cited in: Pubmed; PMID 70475924.

28. Anonymous. Laboratory evidence of human viral and selected nonviral infections in Canada in 1985. CMAJ. 1986 Nov 15;135(10):1155-6. eng. Epub 1986/11/15. Cited in: Pubmed; PMID 3768823.

29. Anonymous. Laboratory reports of herpesvirus infections in Canada in 1984. CMAJ. 1986 Feb 1;134(3):249-51. eng. Epub 1986/02/01. Cited in: Pubmed; PMID 3002575.

30. Anonymous. Sexually transmitted disease in Canada in 1984. Canadian Medical Association Journal. 1986;134(8):919-920. Cited in: Pubmed; PMID 16013795.

31. Anonymous. Laboratory reports of human viral and selected non-viral agents in Canada--1992. Can Commun Dis Rep. 1993 Nov 30;19(22):188-93. eng

fre. Epub 1993/11/30. Cited in: Pubmed; PMID 8111342.

32. Auger N, Quach C, Healy-Profitos J, Lowe AM, Arbour L. Congenital microcephaly in Quebec: Baseline prevalence, risk factors and outcomes in a large cohort of neonates. Archives of Disease in Childhood: Fetal and Neonatal Edition. 2018 March;103(2):F167-F172. Cited in: Pubmed; PMID 617544311.

33. Bodsworth N, Bloch M, McNulty A, Denham I, Doong N, Trottier S, Adena M, Bonney MA, Agnew J. 2-day versus 5-day famciclovir as treatment of recurrences of genital herpes: results of the FaST study. Sex Health. 2008 Sep;5(3):219-25. eng. Epub 2008/09/06. doi:10.1071/sh08013. Cited in: Pubmed; PMID 18771636.

34. Delva MD, McSherry JA. Herpes genitalis in a student population. J Fam Pract. 1984 Mar;18(3):397-400. eng. Epub 1984/03/01. Cited in: Pubmed; PMID 6699579.

35. Diaz-Mitoma F, Sibbald RG, Shafran SD, Boon R, Saltzman RL. Oral famciclovir for the suppression of recurrent genital herpes: a randomized controlled trial. Collaborative Famciclovir Genital Herpes Research Group. Jama. 1998 Sep 9;280(10):887-92. eng. Epub 1998/09/18. doi:10.1001/jama.280.10.887. Cited in: Pubmed; PMID 9739972.

36. Eisler D, McNabb A, Mak A, Krajden M, Petric M, Tang P. Development of a real-time PCR assay for herpes simplex virus types 1 and 2 and varicella-zoster virus. Canadian Journal of Infectious Diseases and Medical Microbiology. 2010 May;SA):27A. Cited in: Pubmed; PMID 70468103.

37. Embil JA, Garner JB, Pereira LH. Association of cytomegalovirus and herpes simplex virus infections of the cervix in four clinic populations. Sexually Transmitted Diseases. 1985;12(4):224-228. Cited in: Pubmed; PMID 16214008.

38. Embil JA, Stephens RG, Manuel FR. Prevalence of recurrent herpes labialis and aphthous ulcers among young adults on six continents. Can Med Assoc J. 1975 Oct 4;113(7):627-30. eng. Epub 1975/10/04. Cited in: Pubmed; PMID 1181018.

39. Ernst LM, Bockoven C, Freedman A, Wang V, Pellerite M, Wylie TN, Wylie KM. Chronic villitis of unknown etiology: Investigations into viral pathogenesis. Placenta. 2021 April;107:24-30. Cited in: Pubmed; PMID 2011329838.

40. Ezzat HM, Cheung MC, Hicks LK, Boro J, Montaner JSG, Lima VD, Harris M, Leitch HA. Incidence, predictors and significance of severe toxicity in patients with human immunodeficiency virus-associated Hodgkin lymphoma. Leukemia and Lymphoma. 2012 December;53(12):2390-2396. Cited in: Pubmed; PMID 365983945.

41. Gesink D, Wang S, Guimond T, Kimura L, Connell J, Salway T, Gilbert M, Mishra S, Tan D, Burchell AN, Brennan DJ, Logie CH, Grace D. Conceptualizing Geosexual Archetypes: Mapping the Sexual Travels and Egocentric Sexual Networks of Gay and Bisexual Men in Toronto, Canada. Sexually Transmitted Diseases. 2018 01 Jun;45(6):368-373. Cited in: Pubmed; PMID 622354662.

42. Gill MJ, Arlette J, Buchan K. Herpes simplex virus infection of the hand. A profile of 79 cases. Am J Med. 1988 Jan;84(1):89-93. eng. Epub 1988/01/01. doi:10.1016/0002-9343(88)90013-7. Cited in: Pubmed; PMID 2827469.

43. Guibinga GH, Coutlée F, Kessous A, Hankins C, Lapointe N, Richer G, Tousignant J. Detection of a transforming fragment of herpes simplex virus type 2 in clinical specimens by PCR. The Canadian Women's HIV Study Group. J Clin Microbiol. 1996 Jul;34(7):1654-9. eng. Epub 1996/07/01. doi:10.1128/jcm.34.7.1654-1659.1996. Cited in: Pubmed; PMID 8784564.

44. Gully PR, Cloutier Fisher D, Pless R, Herbert C. How well do family physicians manage sexually transmitted diseases? Canadian Family Physician. 1995;41(NOV.):1890-1896. Cited in: Pubmed; PMID 25369932.

45. Hamandi B, Husain S, Grootendorst P, Papadimitropoulos EA. Clinical and microbiological epidemiology of early and late infectious complications among solid-organ transplant recipients requiring hospitalization. Transplant International. 2016 01 Sep;29(9):1029-1038. Cited in: Pubmed; PMID 611936010.

46. Hammerberg O, Watts J, Chernesky M, Luchsinger I, Rawls W. An outbreak of herpes simplex virus type 1 in an intensive care nursery. Pediatr Infect Dis. 1983 Jul-Aug;2(4):290-4. eng. Epub 1983/07/01. doi:10.1097/00006454-198307000-00007. Cited in: Pubmed; PMID 6310534.

47. Hankins CA, Gendron S, Handley MA, Richard C, Tung MT, O'Shaughnessy M. HIV infection among women in prison: an assessment of risk factors using a nonnominal methodology. Am J Public Health. 1994 Oct;84(10):1637-40. eng. Epub 1994/10/01. doi:10.2105/ajph.84.10.1637. Cited in: Pubmed; PMID 7943484.

48. Hockin JC. A survey of sexually transmitted diseases diagnosed by physicians in Newfoundland. Canadian Journal of Public Health. 1985;76(1):30-32. Cited in: Pubmed; PMID 15139923.

49. Jassem A, Krajden M, Getman D, Hovey P, Hentzen C, Barakat N, Jiang A. Comparative evaluation of the Aptima HSV 1&2 assay and a lab developed real-time PCR test for detection of HSV-1 and HSV-2 viruses. Journal of Clinical Virology. 2016 September;82(Supplement 1):S31. Cited in: Pubmed; PMID 613190929.

50. Kariyawasam R, Lau R, Eshaghi A, Patel SN, Sider D, Gubbay JB, Boggild AK. Spectrum of viral pathogens in blood of malaria-free Ill travelers returning to Canada. Emerging Infectious Diseases. 2016 May;22(5):854-861. Cited in: Pubmed; PMID 609995808.

51. Kaspirkova J, Gomolcakova B, Ondic O, Michal M. The assessment of potential biomarkers of disease progression in high grade squamous cervical lesions-promoter methylation analysis of selected tumour-suppressor genes and viral/microbial co-infection. Laboratory Investigation. 2016 February;1):290A.

52. Kolski H, Ford-Jones EL, Richardson S, Petric M, Nelson S, Jamieson F, Blaser S, Gold R, Otsubo H, Heurter H, MacGregor D. Etiology of acute childhood encephalitis at The Hospital for Sick Children, Toronto, 1994-1995. Clin Infect Dis. 1998 Feb;26(2):398-409. eng. Epub 1998/03/21. doi:10.1086/516301. Cited in: Pubmed; PMID 9502462.

53. Logie CH, Navia D, Loutfy MR. Correlates of a lifetime history of sexually transmitted infections among women who have sex with women in Toronto, Canada: results from a cross-sectional internet-based survey. Sex Transm Infect. 2015 Jun;91(4):278-83. eng. Epub 2014/12/06. doi:10.1136/sextrans-2014-051745. Cited in: Pubmed; PMID 25477474.

54. McClymont E, Coutlee F, Lee M, Albert A, Walmsley S, Lipsky N, Ogilvie G, Tan D, Money D. HSV-2 serostatus and HPV incidence, persistence, and precancerous lesions in a cohort of HPV-vaccinated women living with HIV. Sexually Transmitted Infections. 2019 July;95(Supplement 1):A350. Cited in: Pubmed; PMID 629059836.

55. McSherry JA. Silent genital herpes simplex infection among asymptomatic female college students. J R Soc Health. 1986 Jun;106(3):96-7. eng. Epub 1986/06/01. doi:10.1177/146642408610600309. Cited in: Pubmed; PMID 3090261.

56. Nicolle LE, Minuk GY, Postl B. Cross-sectional seroepidemiologic study of the prevalence of cytomegalovirus and herpes simplex virus infection in a Canadian Inuit (Eskimo) community. Scandinavian Journal of Infectious Diseases. 1986;18(1):19-23. Cited in: Pubmed; PMID 16135618.

57. Piret J, Gagne N, Perron S, Desormeaux A, Tremblay MJ, Gourde P, Omar RF, Bergeron MG. Thermoreversible gel as a candidate barrier to prevent the transmission of HIV-1 and herpes simplex virus type 2. Sexually Transmitted Diseases. 2001;28(8):484-491. Cited in: Pubmed; PMID 32730298.

58. Plotnikoff KM, Ogilvie GS, Smith L, Donken R, Pedersen HN, Samji H, Grennan T. Factors associated with interest in bacterial sexually transmitted infection vaccines at two large sexually transmitted infection clinics in British Columbia, Canada. Sexually Transmitted Infections. 2020 01 Nov;96(7):494-500.

59. Rawls WE, Lavery C, Marrett LD, Clarke EA, Adam E, Melnick JL, Best JM, Kraiselburd E, Benedet LJ, Brenes MM, et al. Comparison of risk factors for cervical cancer in different populations. Int J Cancer. 1986 Apr 15;37(4):537-46. eng. Epub 1986/04/15. doi:10.1002/ijc.2910370411. Cited in: Pubmed; PMID 3007372.

60. Redmond M, Schumacher B. Study of the abnormal cervical-vaginal cytology of sexually active young women living within the Waterloo region. Can J Public Health. 1990 May-Jun;81(3):235-6. eng. Epub 1990/05/01. Cited in: Pubmed; PMID 2361216.

61. Shukalek CB, Lee B, Fathima S, Chu A, Fonseca K, Somayaji R. Comparative Analysis of Molecular and Serologic Testing for Primary Syphilis: A Population-Based Cohort Study. Front Cell Infect Microbiol. 2021;11:579660. eng. Epub 2021/05/11. doi:10.3389/fcimb.2021.579660. Cited in: Pubmed; PMID 33968792.

62. Tremblay C, Coutlée F, Weiss J, Guibinga GH, Hankins C, Lapointe N. Evaluation of a non-isotopic polymerase chain reaction assay for detection in clinical specimens of herpes simplex virus type 2 DNA. Canadian Women's HIV Study Group. Clin Diagn Virol. 1997 May;8(1):53-62. eng. Epub 1997/05/01. doi:10.1016/s0928-0197(97)00012-3. Cited in: Pubmed; PMID 9248658.

63. Tremblay MA, Rodrigue MA, Deschenes L, Boivin G, Longtin J. Cytomegalovirus quantification in plasma with Abbott RealTime CMV and Roche Cobas Amplicor CMV assays. Journal of Virological Methods. 2015 December 01;225:1-3. Cited in: Pubmed; PMID 605947698.

64. Vynograd N, Vynograd I, Sosnowski Z. A comparative multi-centre study of the efficacy of propolis, acyclovir and placebo in the treatment of genital herpes (HSV). Phytomedicine. 2000 Mar;7(1):1-6. eng. Epub 2000/04/27. doi:10.1016/s0944-7113(00)80014-8. Cited in: Pubmed; PMID 10782483.

65. Wilson GAR, Weber JM, Paulson E. Laboratory reports of human viral and selected nonviral agents in Canada - 1993. Cmaj. 1995;153(1):51-53. Cited in: Pubmed; PMID 25223007.

66. Zuckerman RA, Limaye AP. Varicella zoster virus (VZV) and herpes simplex virus (HSV) in solid organ transplant patients. American Journal of Transplantation. 2013;13(SUPPL. 3):55-66. Cited in: Pubmed; PMID 372366306.

67. Kaul R, Chieza L, Rebbapragada A, Loutfy M, Tharao W, Saunders M, Huibner S, Liu J, Remis RS, Green-Walker LA. Prevalence and associations of infection by herpes simplex virus type 2 (HSV-2) in African-caribbean women in Toronto. Canadian Journal of Infectious Diseases and Medical Microbiology. 2010 Summer;SB):12B. Cited in: Pubmed; PMID 70474285.

68. Liu J, Remis RS, Loutfy M, Tharao W, Rebbapragada A, Robinette J, Halpenny R, Grennan T, Brunetta J, Smith G, Reko T, Kaul R. Prevalence and correlates of sexually transmitted co-infections in HIV-positive and HIV-negative men who have sex with men in Toronto. Canadian Journal of Infectious Diseases and Medical Microbiology. 2014 March-April;SA):73A-74A. Cited in: Pubmed; PMID 71972629.

69. Shannon B, Yi TJ, Thomas-Pavanel J, Chieza L, Janakiram P, Saunders M, Tharao W, Huibner S, Remis R, Rebbapragada A, Kaul R. Impact of asymptomatic herpes simplex virus type 2 infection on mucosal homing and immune cell subsets in the blood and female genital tract. Journal of Immunology. 2014 01 Jun;192(11):5074-5082. Cited in: Pubmed; PMID 373143300.

70. Tan DH, Kaul R, Raboud JM, Walmsley SL. No impact of oral tenofovir disoproxil fumarate on herpes simplex virus shedding in HIV-infected adults. AIDS. 2011 Jan 14;25(2):207-10. eng. Epub 2010/12/15. doi:10.1097/QAD.0b013e328341ddf7. Cited in: Pubmed; PMID 21150556.

71. Tan DH, Raboud JM, Kaul R, Walmsley SL. Antiretroviral therapy is not associated with reduced herpes simplex virus shedding in HIV coinfected adults: an observational cohort study. BMJ Open. 2014 Jan 24;4(1):e004210. eng. Epub 2014/01/28. doi:10.1136/bmjopen-2013-004210. Cited in: Pubmed; PMID 24464523.

72. Tan DH, Raboud JM, Szadkowski L, Yi TJ, Shannon B, Kaul R, Liles WC, Walmsley SL. Herpes simplex virus type 2 serostatus is not associated with inflammatory or metabolic markers in antiretroviral therapy-treated HIV. AIDS Res Hum Retroviruses. 2015 Mar;31(3):276-81. eng. Epub 2014/11/18. doi:10.1089/aid.2014.0183. Cited in: Pubmed; PMID 25399537.

73. Tan DH, Szadkowski L, Raboud JM, Yi TJ, Shannon B, Kaul R, Liles WC, Walmsley S. Effect of intercurrent infections and vaccinations on immune and inflammatory biomarkers among HIV-infected adults on suppressive antiretroviral therapy. Canadian Journal of Infectious Diseases and Medical Microbiology. 2015 March-April;SB):19B-20B. Cited in: Pubmed; PMID 71971229.

74. Jessamine AG. Laboratory reports of herpesvirus infections in Canada in 1983. Canadian Medical Association Journal. 1985;132(1):49-50. Cited in: Pubmed; PMID 15198684.

75. Government of Prince Edward Island. PEI CPHO Report and Health Trends. 2014 April 25,. Available from: <https://www.princeedwardisland.ca/en/topic/health-pei>.

76. Public Health Agency of Canada. 2004 Canadian Sexually Transmitted Infections Surveillance Report. 2007 April 25,. Available from: <https://www.canada.ca/en/public-health/se>.

77. Li X, Kim PH-JK, Gilbert M. Trends in Herpes Simplex Virus Cases in British Columbia, 1992-2006. Citeseer; 2008.
